# *i*LoF: An intelligent Lab on Fiber Approach for Human Cancer Single-Cell Type Identification

**DOI:** 10.1038/s41598-020-59661-5

**Published:** 2020-02-21

**Authors:** Joana S. Paiva, Pedro A. S. Jorge, Rita S. R. Ribeiro, Meritxell Balmaña, Diana Campos, Stefan Mereiter, Chunsheng Jin, Niclas G. Karlsson, Paula Sampaio, Celso A. Reis, João P. S. Cunha

**Affiliations:** 1 INESC TEC - INESC Technology and Science, Porto, Portugal; 2 Physics and Astronomy Department, Faculty of Sciences, University of Porto, Porto, Portugal; 3 Faculty of Engineering, University of Porto, Porto, Portugal; 4 i3s - Instituto de Investigação e Inovação em Saúde, Universidade do Porto, Porto, Portugal; 5 IPATIMUP - Institute of Molecular Pathology and Immunology, University of Porto, Porto, Portugal; 6 Department of Medical Biochemistry and Cell Biology, Institute of Biomedicine, Sahlgrenska Academy, University of Gothenburg, Gothenburg, Sweden; 7 IBMC - Instituto de Biologia Molecular e Celular, Universidade do Porto, Porto, Portugal; 8 Instituto de Ciências Biomédicas Abel Salazar, University of Porto, Porto, Portugal; 9 Faculty of Medicine of the University of Porto, Porto, Portugal; 10 Present Address: 4DCell, Paris, France; 11 Present Address: IMBA, Institute of Molecular Biotechnology of the Austrian Academy of Sciences, Vienna BioCenter Campus, 1030 Vienna, Austria

**Keywords:** Computational biophysics, Tumour biomarkers, Prognostic markers, Applied optics

## Abstract

With the advent of personalized medicine, there is a movement to develop “smaller” and “smarter” microdevices that are able to distinguish similar cancer subtypes. Tumor cells display major differences when compared to their natural counterparts, due to alterations in fundamental cellular processes such as glycosylation. Glycans are involved in tumor cell biology and they have been considered to be suitable cancer biomarkers. Thus, more selective cancer screening assays can be developed through the detection of specific altered glycans on the surface of circulating cancer cells. Currently, this is only possible through time-consuming assays. In this work, we propose the “intelligent” Lab on Fiber (*i*LoF) device, that has a high-resolution, and which is a fast and portable method for tumor single-cell type identification and isolation. We apply an Artificial Intelligence approach to the back-scattered signal arising from a trapped cell by a micro-lensed optical fiber. As a proof of concept, we show that *i*LoF is able to discriminate two human cancer cell models sharing the same genetic background but displaying a different surface glycosylation profile with an accuracy above 90% and a speed rate of 2.3 seconds. We envision the incorporation of the *i*LoF in an easy-to-operate microchip for cancer identification, which would allow further biological characterization of the captured circulating live cells.

## Introduction

Recent research trends on healthcare point out to the movement to develop “smart” micro-tools to allow better personalized diagnostic and therapeutic approaches^1–3^. Considering that current medicine and biotechnology attempts are converging to novel methodologies at the micro (e.g., cancer cells detection) and nano scales (e.g., cancer-related extracellular vesicles detection), an effort towards the development of these “intelligent” microdevices with multifunctionalities is required^3^. In this regard, optical fiber tools - for example, Optical Fiber Tweezers (OFT)^1,4,5^ - have emerged as suitable candidates thanks to their flexibility, small size and chemical inertness, which contributes to the advent of a novel concept of “Lab on Fiber” (LoF) devices^2^. The fruitful application of these optical-based microdevices in cancer screening has been envisioned as straightforward^1,2^. However, the high degree of heterogeneity among cancer subtypes must be taken into consideration^6,7^. This heterogeneity is mainly due to both cellular and microenvironmental factors, such as alterations in cellular glycosylation^8^. In particular, the selective detection of specific cancer-associated glycoforms expressed at the surface of circulating cancer cells could increase the specificity of cancer biomarker assays and therapeutic approaches^8–10^. In fact, tumor heterogeneity is considered to be a major barrier to an effective cancer diagnosis and treatment^6,7^. Recent evidence has shown that glycans can determine the acquisition of certain cellular features controlling tumor growth and progression^8,11,12^. For example, shorter truncated *O*-glycans are considered to be predictive markers of poor prognosis in certain cancers^8,13^. These alterations are currently only possible to detect through complex and time-consuming methods, such as mass spectrometry and affinity assays^9,10,14^. Consequently, an effort to develop novel micro optical “intelligent” devices with high sensitivity is required. Considering the wide range of Artificial Intelligence (AI) decision support algorithms, we postulate that conventional optical fiber tools could be converted into *i*LoF devices, which would be able to immobilize and classify cancer cells alterations with high inter-cell similarity degrees.

Even though the development of OFT is a growing field, only a limited number of options are available for simultaneous cell trapping and sensing^1,2,5,15^. Plasmonic fiber tweezers are good candidates for such an hybrid task because, beyond trapping, they are sensitive to tiny changes of the surrounding environmental refractive index and can then provide some additional information about their target^16^. However, plasmonic devices often require a relatively complex multi-step fabrication process. Additionally, recent evidence has shown that their higher refractive index sensitivity can interfere with their trapping capability^16^.

As an alternative to fiber-based solutions, scattering-based techniques (e.g., Raman spectroscopy, flow cytometry)^17,18^ have also been widely used for cell characterization. In fact, the amount of light that is scattered by a cell is still considered to be among the gold-standard techniques for cell characterization^17,18^. Flow cytometry has been considered to be the most adequate technique for studying cellular viability and morphological measurements^18^. However, flow cytometers are based on bulky and expensive equipment (comprising more than 10 lasers and highly sensitive photodetectors), and they require the analysis of both scattered and fluorescence signals^18^. Additionally, flow cytometry is a multi-event detection system, providing multiparametric information of several particles flowing per second^18^. Unlike flow cytometers, "intelligent” fiber tweezers provide meaningful information of an individualized target particle - that is stably trapped during the measurements.

We have developed an AI method based on the analysis of laser back-scattered signals of trapped cells using spherical lensed optical fiber tips to identify human cancer cells that only differ in their surface glycosylation. This approach is based on the immobilization of the cell under test through a touchless optical trapping force exerted by the polymeric lens on the top of the optical fiber and the simultaneous acquisition of the back-scattered signal arising from the trapped cell. We validated our method named *i*LoF (*intelligent* Lab on Fiber) by subjecting it to a human gastric carcinoma cell line that is genetically modified to over-express the ST6GalNAc1 enzyme and to the corresponding control cell line. The ST6GalNAc1 enzyme is responsible for the expression of the STn antigen, which is a well-established tumor derived carbohydrate antigen associated to metastasis and poor prognosis of cancer patients^19,20^. After applying a robust evaluation scheme, including more than 29,000 independent test runs and a 4-class detection experiment (including the distinction between these two cancer cell models, the condition of “No cell trapped” and of one trapped polystyrene microsphere, the known control), the *i*LoF showed overall accuracy and F-Measure performance values of 0.93 and 0.85, respectively. It was also characterized by a Speed Rate (SR) of approximately 2.3 seconds for 100% of detection accuracy. This high-resolution single-cell characterization method could be embedded into microdevices with innovative attributes. Possible use-case scenarios include subtype identification of circulating live cancer cells, leading to more personalized therapies, or its earlier assessment.

## Results

### Optical trapping of cancer cells

**Figure 1:**
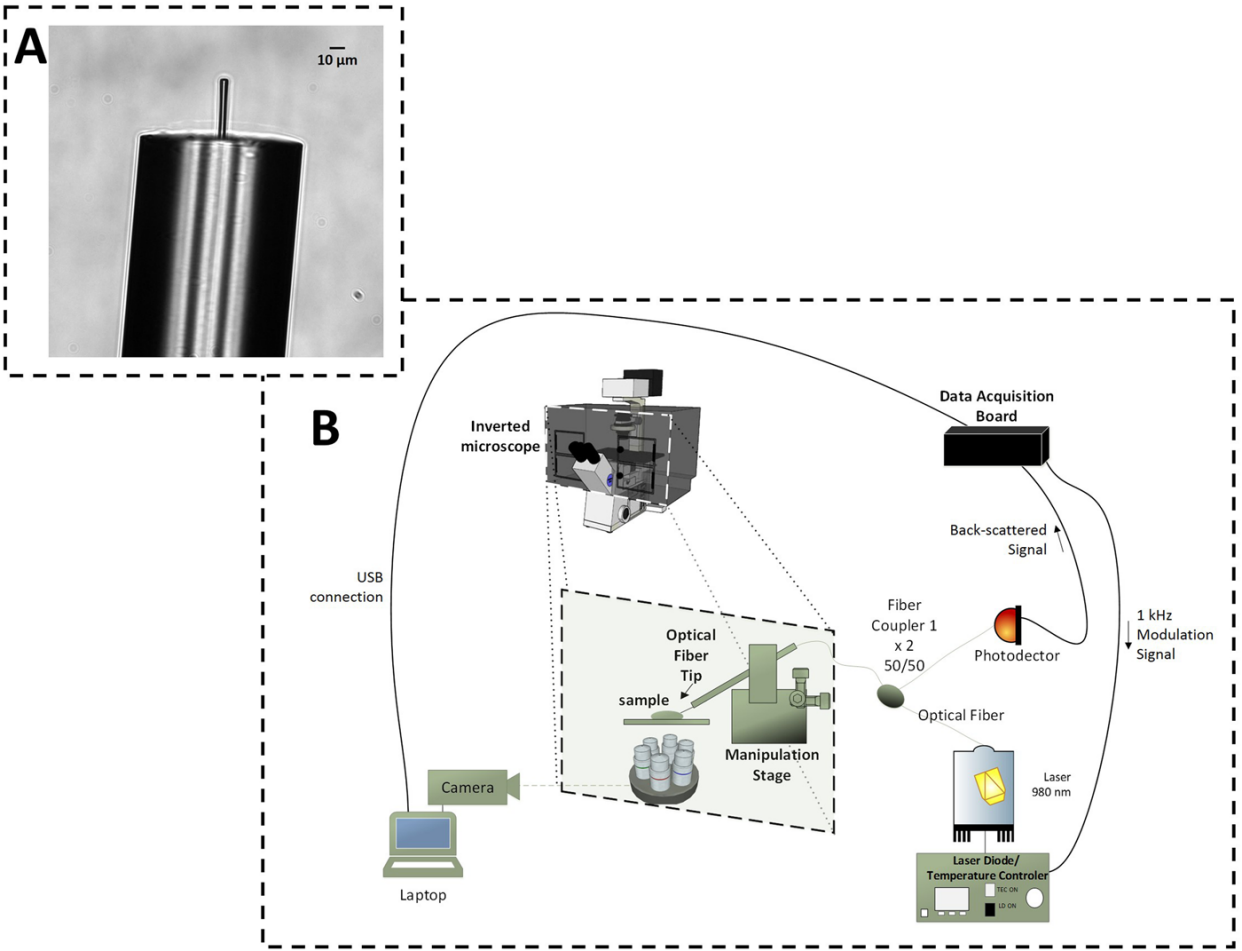
Microscopic image of the polymeric lens on the top of the optical fiber and optical manipulation setup used to trap particles and cancer cells. (Panel A) Bright-field microscopic image of the polymeric lensed optical fiber tip. (Panel B) The setup designed consisted of an inverted microscope connected to additional three subsystems: image acquisition, micromanipulation and signal acquisition modules. Cell/particle samples were maintained within the temperature and atmosphere controlled chamber.

To develop this novel “intelligent” method to simultaneously trap and identify different human cancer cells we first had to fabricate a lensed fiber tip that is able to individually optically-trap such cells with no material contact to minimize cell disturbance. We also had to design an optical setup for scatterers visualization, manipulation and back-scattered signal acquisition (Fig. 1).

The lens-like microstructure that we used to trap cells (Fig. 1(A)) was fabricated on the top of a single mode optical fiber, through a waveguide photo-polymerization method^4,21^ (Online Methods). It is characterized by a spherical geometry, a refractive index of 1.52, a length, base diameter and curvature radius of ≈45 *μ*m, ≈6 *μ*m and ≈3 *μ*m, respectively; and a Numerical Aperture (NA) of 0.5 ≤ NA ≤ 0.6.

An inverted microscope-based setup was therefore designed and mounted to characterize and quantify the optical trapping ability of the proposed microlens on each cell model. The setup consisted of an inverted microscope connected to additional three subsystems: the image acquisition, the micromanipulation and the signal acquisition modules (Fig. 1(B)). The last subsystem was included to acquire the back-scattered signal while the cells were trapped.

**Figure 2:**
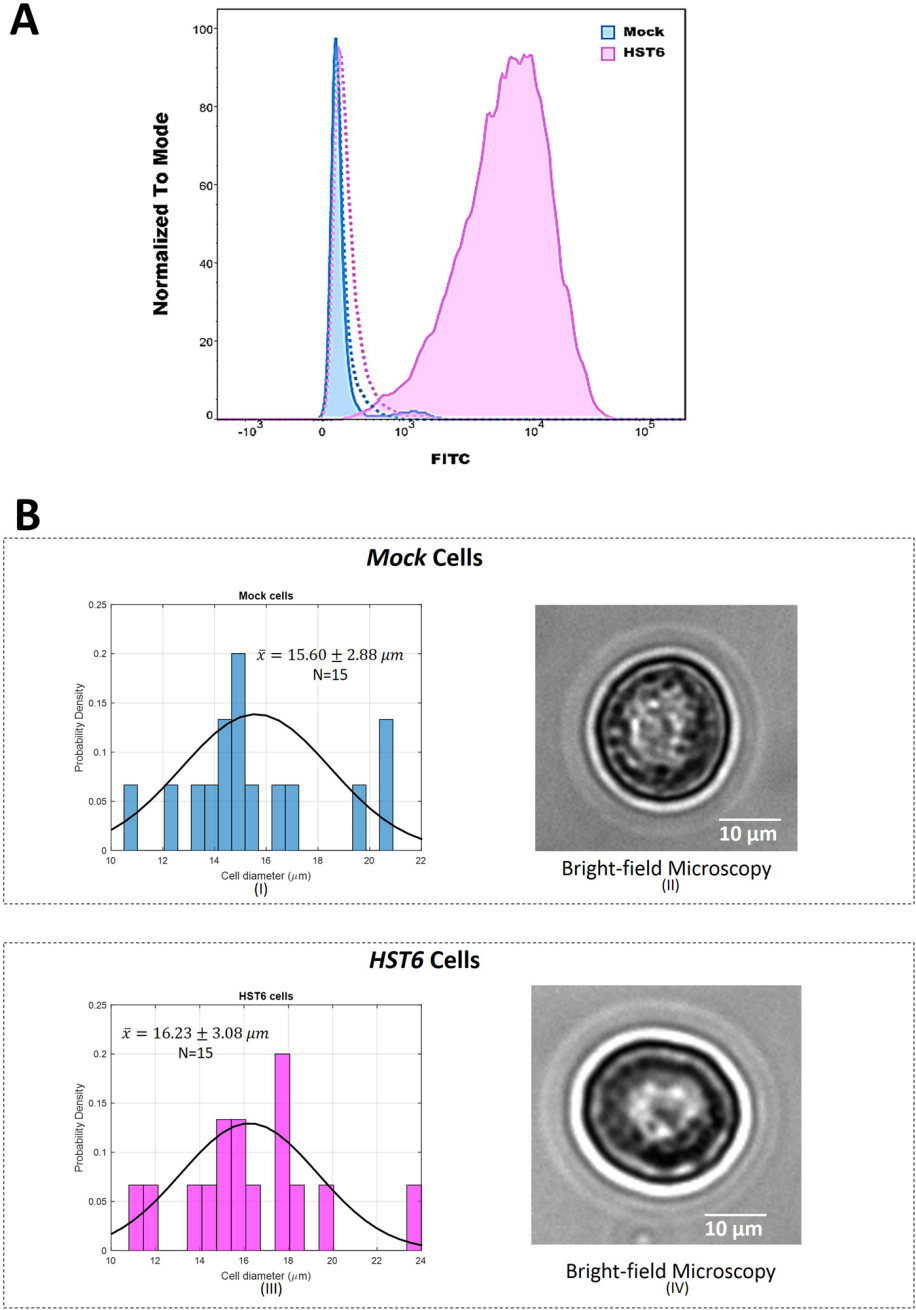
Characterization of the *ST6* gastric cancer cell model. (Panel A) Flow cytometry analysis of STn expression in HST6 cells compared to the *Mock* control cell line. The negative controls are shown in dotted lines. Two independent experiments were performed. (Panel B) (I,III) Probability Density Histograms showing cell diameter distribution and corresponding normal curve fit for (I) *Mock* and (III) *HST6* cells (*P*_*Shapiro*–*Wilk* *Normality* *test*_ > 0.05, two tailed). (II,IV) Examples of bright-field microscopic images of of a (II) *Mock* cell and a (IV) *HST6* cell. There was no significant difference between cell type diameters (*P*_*Student*7D1*t*–*test*_ > 0.05; unpaired, two tailed; *n* = 15).

The two selected cell lines to test our method were derived from the gastric cancer cell line MKN45: *HST6*, which was genetically modified to present truncated *O*-glycans at their surface, due to the over-expression of the ST6GalNAc1 sialyltransferase - and *Mock* - the corresponding control cells transfected with the empty vector^19^. The overexpression of the *α*2,6-sialyltransferase ST6GalNAc1 resulted in a different cellular glycosylation profile, showing the *de novo* STn expression (Fig. 2(A)). To further characterize this model, we have performed glycomic analyses. We identified 18 *N*-glycan structures in both *HST6* transfected cells and *Mock*, covering pauci-mannose, oligo-mannosidic, hybrid and complex *N*-glycans (Table S1, Supplementary Material). The same *N*-glycan structures were identified in both cell lines, and only limited quantitative differences were detected, indicating no effect of ST6GALNAC1 overexpression on the N-glycome. The *O*-glycomic analysis revealed 19 *O*-glycan structures, including STn, core 1, core 2 and core 3 structures (Table S2), Supplementary Material). Most structures identified had terminal sialic acids with core 2 structures being the most elongated. *HST6* overexpressing cells showed in accordance with the previous flow cytometry results a significant increase in STn.

**Figure 3:**
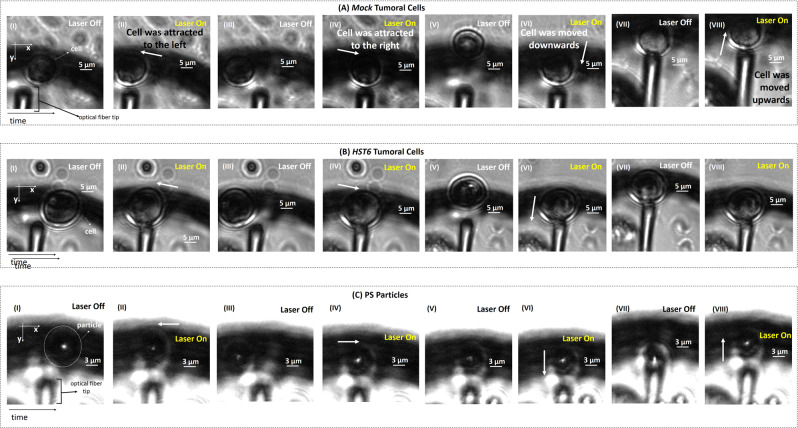
Snapshots showing the trapping ability of the proposed spherical lenses on top of fibers for (**A**) a *Mock* tumoral cell, (**B**) a *HST6* tumoral cell and (**C**) a Polystyrene particle as a target. (**A**–**C**)-I - The optical fiber tip is displaced towards the left (−*x* direction) (with the laser off) in relation to the target. (**A**–**C**)-II - The laser is turned on and the particle is attracted to the equilibrium position (trapping position) where it remains immobilized. (**A**–**C**)-III - The laser is again turned off and the fiber tip displaced towards the opposite transversal direction (towards the right, +*x* direction). (**A**–**C**)-IV - After the laser is turned on, the particle is displaced towards the right due to optical trapping forces. (**A**–**C**)-V - In order to study the longitudinal trapping forces profile for each particle type, the fiber tip is moved towards +*y* direction (down) with the laser off. (**A**–**C**)-VI - Particles are pushed after the laser is turned on. (**A**–**C**)-VII - The laser is turned off and the fiber tip is now moved along the longitudinal direction (towards −*y*, up). (**A**–**C**)-VIII - Particles are pulled due to optical trapping, excepting *HST6* cells (cell movement due to trapping effects along *-y* direction are almost imperceptible, since the axial contribution of the gradient force to the total trapping force is negligible, in comparison with the transversal component of the gradient force, which plays the major role in the trapping phenomena).

The two cell models were subjected to morphological analysis and no significant differences were displayed between them (Fig. 2(B)). The profile of trapping forces exerted by the fabricated microlens was characterized by considering three types of particles: cancer cells *Mock* and *HST6*; and 8 *μ*m diameter polystyrene (PS) synthetic microspheres (Supplementary Table S3). After the described setup was correctly mounted, a drop of each solution containing the particles to analyze (Supplementary Table S3) was placed over a 35 mm dish, and the lensed fiber tip was inserted into this sample at an inclination angle of 50^°^. Multiple snapshots of the microlens trapping each particle were acquired. Trapping force measurements were then performed through the *Drag Force* method (Online Methods). PS microparticles were included as “known controls”, because our previous studies showed that PS microparticles can be successfully trapped using this type of lens. All of the analyzed microparticles were successfully trapped in two dimensions (2D), as depicted in Fig. 3. Confinement in the third dimension was ensured by the presence of a glass slide surface. Three dimensional optical trapping was not verified, because it usually requires higher power densities (stronger focusing), which can eventually damage the cells. Although all of the particles were successfully trapped along all the transversal directions (left and right, up and down, having the trapping equilibrium point as reference), the displacement towards −*y* direction was almost insignificant for *HST6* cells (Fig. 3B-VII,VIII). Thus, the trapping forces were only compared among particles by considering the transversal displacements along the *xx* axis, relative to the propagation direction of the laser beam.

The resultant trapping forces exerted on each particle result from the sum of two components: the scattering and gradient forces^22^, both of which are dependent on the diameter of the trapped particle^22^. In this particular case, a single beam is used for 2D trapping. Thus, the transversal and longitudinal particle displacements relative to waveguide position were due to the transversal and axial components of the gradient force, respectively. According to our previous studies where the trapping forces profile exerted by this kind of lenses was theoretically characterized^4,23,24^, the axial contribution of the gradient force can be usually considered negligible, because the transversal component of the gradient force plays the major role in the trapping phenomena. Thus, it is comprehensible that axial particles displacement due to optical trapping was weaker in comparison with the longitudinal component, leading to an almost imperceptible *HST6* cell displacement towards −*y* direction (Fig. 3B-VII,VIII).

**Figure 4:**
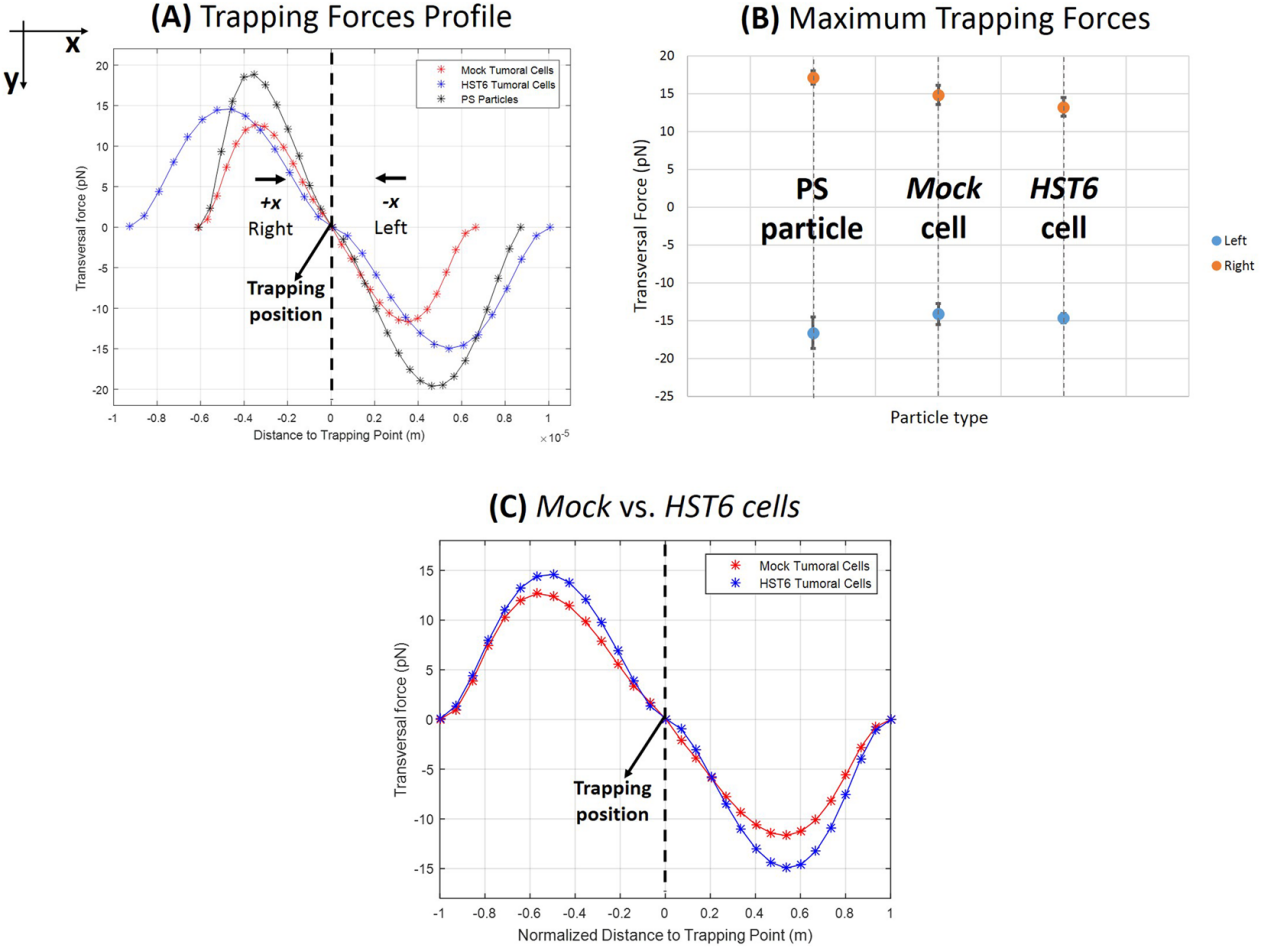
Description of transversal trapping forces exerted by the fabricated polymeric tip on *Mock* and *HST6* tumoral cells and polystyrene particle. (**A**) Forces profile acting on each type of microparticle according to its position relatively to the trapping point (equilibrium position where each particle is stably trapped and the resultant of the forces acting on it is approximately null). The left part of the curves (corresponding to particle positions at the left of the equilibrium point) describe trapping forces profile when the particle is displaced towards the right (towards the +*x* direction) due to optical trapping. The right-hand side of the curves (corresponding to positions at the right of the trapping point) represent trapping forces exerted on the particle when it is moved towards −*x* direction (to the left). (**B**) Average maximum trapping forces exerted on *Mock*, *HST6* cells and polystyrene particles, for left (blue) and right (orange) particle displacements due to optical trapping among the three displacements performed for each direction. (**C**) Comparison of forces exerted on *Mock* and *HST6* cells considering distance points to trapping force normalized to the maximum displacement achieved by each cell due to optical trapping for one of the three displacements recorded for optical force analysis ($P > \frac{0.05}{n},n=30$; Student T-test for independent samples with correction for multiple comparisons).

Maximum trapping force magnitude values for each type of particle are depicted in Fig. 4(A,C)). The target submitted to the strongest trapping force was found to be the synthetic particle. This is an expected outcome - considering that the gradient force increases with the product between the radius of the particle and the difference between its refractive index and the media, which is defined as the "optical size” of the particle^22^. In fact, although the cancer cells had diameters higher than the polystyrene particle, the latter was characterized by a refractive index of 1.57^25^, while human cancer cells were characterized by values within 1.36–1.37^26^, which are very close to the media refractive index (PBS, 1.36). We therefore infer that the refractive index difference between biological and synthetic particles had surpassed the particles’ size differences.

The trapping force measurement assay showed that it is possible to stably immobilize both types of cancer cell models using the fabricated microlens. This ensured that any type of signal acquired from trapped cells would be mostly comprised of back-scattered photons from the cell, minimizing noisy information derived from random particle motion in the solution (e.g., Brownian motion).

### High-resolution artificial intelligence-based cancer cells identification

A novel Artificial Intelligence (AI) method was developed to discriminate different cancer cell models, based on time- and frequency-domain parameters derived from short-term back-scattered signal portions from an optically-trapped single-cell (Online Methods). A distinction problem involving four classes (“No particle trapped”; “*Mock* cancer cell trapped”; “*HST6* cancer cell trapped” and a “PS microsphere trapped” - known control) was therefore considered for training and testing the *i*LoF method. The inclusion of the “No Particle” class is relevant for training the algorithm because it can continuously verify if a given cell/particle was optically-trapped or not. A total of 15 cancer cells from each model and 10 polystyrene particles were used in this experiment (see Supplementary Table S3). Note that a number between 500 and 5000 training data samples provided from 20–100 different entities (patients, cells, organs, etc) is frequently reported in several state-of-the-art studies about machine learning-based algorithms for diagnosis and prognosis, mainly focused on cell analysis and cell type classification^27–30^.

**Figure 5:**
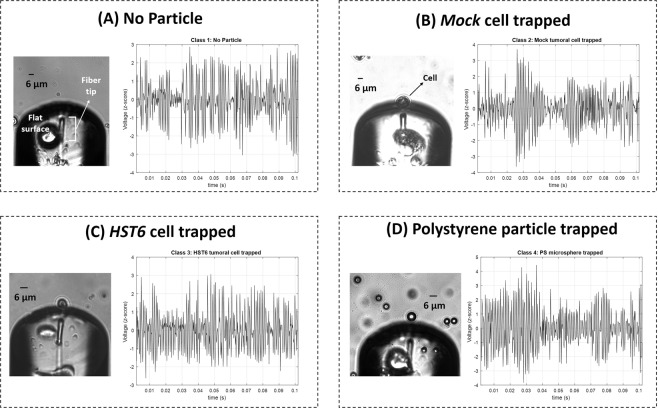
Sketches of back-scattered signal portions and bright-field microscopic images acquired for the different particles trapped: (**A**) no particle; (**B**) *Mock* cell; (**C**) *HST6* cell and (**D**) polystyrene particle.

After collecting enough information for force analysis, each cell/particle was immobilized using the fiber tool as depicted in Supplementary Fig. S2 during 80-seconds^15^, for back-scattered signal acquisition. Part of the light scattered by the particle was collected by the microlens on the top of the optical fiber and recorded by the photodetector. After acquisition, back-scattered signals were processed according to the scheme of Supplementary Fig. S3 (Online Methods). Our final dataset was comprised of 2-seconds portions of back-scattered signal acquired for each particle (Supplementary Table S4). After signal processing (Fig. S3(1–3)), the visual aspect of the resultant signal portions for each class is depicted in Fig. 5. It is clear that signal differences between the type of particles are not visible to the naked eye. Then, we trained a supervised learning-based algorithm, the Random Forests^31,32^, to correctly identify the type of particle trapped, taking into account the information provided from the features set enumerated in Table 1^15^, characterizing each signal portion.

**Table 1:** Summary of the the 54 features used in the classification.

Type	Group	Number	Feature/Parameter
Time Domain	Time Domain Statistics	1	Standard Deviation (SD)
		2	Root Mean Square (RMS)
		3	Skewness (Skew)
		4	Kurtosis (Kurt)
		5	Interquartile Range (IQR)
		6	Entropy (E)
	Time Domain Histogram	7	*μ*_*Nakagami*_
		8	*ω*_*Nakagami*_
Frequency Domai	Discrete Cosine Transform (DCT)	9	1st Coefficient (*E*_*DCT*_[*l*^1^])
		10	2nd Coefficient (*E*_*DCT*_[*l*^2^])
		11	3rd Coefficient (*E*_*DCT*_[*l*^3^])
		12	4th Coefficient (*E*_*DCT*_[*l*^4^])
		13	5th Coefficient (*E*_*DCT*_[*l*^5^])
		14	6th Coefficient (*E*_*DCT*_[*l*^6^])
		15	7th Coefficient (*E*_*DCT*_[*l*^7^])
		16	8th Coefficient (*E*_*DCT*_[*l*^8^])
		17	9th Coefficient (*E*_*DCT*_[*l*^9^])
		18	10th Coefficient (*E*_*DCT*_[*l*^10^])
		19	11th Coefficient (*E*_*DCT*_[*l*^11^])
		20	12th Coefficient (*E*_*DCT*_[*l*^12^])
		21	13th Coefficient (*E*_*DCT*_[*l*^13^])
		22	14th Coefficient (*E*_*DCT*_[*l*^14^])
		23	15th Coefficient (*E*_*DCT*_[*l*^15^])
		24	16th Coefficient (*E*_*DCT*_[*l*^16^])
		25	17th Coefficient (*E*_*DCT*_[*l*^17^])
		26	18th Coefficient (*E*_*DCT*_[*l*^18^])
		27	19th Coefficient (*E*_*DCT*_[*l*^19^])
		28	20th Coefficient (*E*_*DCT*_[*l*^20^])
		29	21st Coefficient (*E*_*DCT*_[*l*^21^])
		30	22nd Coefficient (*E*_*DCT*_[*l*^22^])
		31	23rd Coefficient (*E*_*DCT*_[*l*^23^])
		32	24th Coefficient (*E*_*DCT*_[*l*^24^])
		33	25th Coefficient (*E*_*DCT*_[*l*^25^])
		34	26th Coefficient (*E*_*DCT*_[*l*^26^])
		35	27th Coefficient (*E*_*DCT*_[*l*^27^])
		36	28th Coefficient (*E*_*DCT*_[*l*^28^])
		37	29th Coefficient (*E*_*DCT*_[*l*^29^])
		38	30th Coefficient (*E*_*DCT*_[*l*^30^])
		39	Number of coefficients that capture 98% of the original signal (*N*_*DCT*_)
		40	Total spectrum Area Under Curve (AUC) (*AUC*_*DCT*_)
		41	Maximum peak amplitude (*Peak*_*DCT*_)
		42	Total spectral power (*P*_*DCT*_)
	Wavelet Packet Decomposition	43	Haar Relative Power 1st level (${E}_{Haar}^{1}$)
		44	Haar Relative Power 2nd level (${E}_{Haar}^{2}$)
		45	Haar Relative Power 3rd level (${E}_{Haar}^{3}$)
		46	Haar Relative Power 4th level (${E}_{Haar}^{4}$)
		47	Haar Relative Power 5th level (${E}_{Haar}^{5}$)
		48	Haar Relative Power 6th level (${E}_{Haar}^{6}$)
		49	Db10 Relative Power 1st level (${E}_{Db10}^{1}$)
		50	Db10 Relative Power 2nd level (${E}_{Db10}^{2}$)
		51	Db10 Relative Power 3rd level (${E}_{Db10}^{3}$)
		52	Db10 Relative Power 4th level (${E}_{Db10}^{4}$)
		53	Db10 Relative Power 5th level (${E}_{Db10}^{5}$)
		54	Db10 Relative Power 6th level (${E}_{Db10}^{6}$)

Particle type performance classification was obtained by considering a highly robust *Leave-One-Out*-based procedure to report performance results as closer as possible to a real scenario^31,33^. According to this scheme, 29,250 independent tests were performed, corresponding to the number of possible combinations between a test set comprised by one particle from each one of the four classes. A training set defined by the remaining particles (Supplementary Table S3; Online Methods). Thus, we ensured that all of the particles that we considered were used to both train and test the algorithm, and that the data used for training the classifier was never involved in the test, considering each *n*^*th*^ evaluation run (Supplementary Fig. S4; Online Methods). The *i*LoF method ensured an average accuracy and F-Measure values of 0.93 ± 0.05(*n* = 29, 250) and 0.85 ± 0.13(*n* = 29, 250), respectively (Table 2). Given that the F-Measure is a harmonic mean of the sensitivity and specificity^31^, and considering that our dataset is unbalanced regarding the number of training/test samples per class (Table S4), we can conclude that the *i*LoF is both sensitive and specific.

**Table 2:** *i*LoF classification performance results for the 4-classes identification problem. ^*^Corresponds to the *n* different combinations for particles ID between training and test sets. Avg - average. SD - standard deviation.

*i*LoF Classification Performance			
Nr. of Evaluation Runs (n)^*^	Train	Test	
29,250	F-Measure (Avg. ± SD)	Accuracy (Avg. ± SD)	F-Measure (Avg. ± SD)
	0.93 ± 0.01	0.93 ± 0.05	0.85 ± 0.13

It is worth mentioning here that the classification algorithm was robust to the inter-class variability (e.g., in particle size). In fact, *Mock* cells diameter ranged between 10.1 and 20.8 *μ*m, while *HST6* cells were characterized by diameters between 11.3 and 23.8 *μ*m. Nevertheless, the *i*LoF method was able to distinguish the two cancer models with an accuracy per class of 0.92 and 0.89 for *Mock* and *HST6* cells, respectively (Supplementary Fig. S6). However and according to what was expected due to their similarity, the mean accuracy per class among the 29,250 runs was lower for cancer cells in comparison with “No particle trapped” and “PS microsphere trapped” classes (Supplementary Fig. S6). Still, the mean accuracy per class values were above 89% for all the classes considered.

### *i*LoF speed detection rate

**Figure 6:**
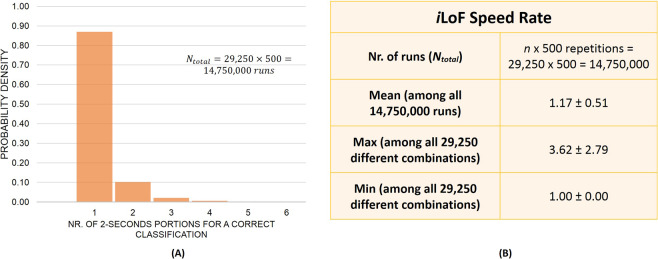
*i*LoF SR. (**A**) Probability density histogram regarding the number of 2-seconds signal portions needed to correctly identify the analyzed cell class, among *n* × 500 = 29, 250 × 500 = 14, 750, 000 independent runs. (**B**) *i*LoF statistics in terms of the number of 2-seconds short-term signal portions needed to correctly identify the particle/cell trapped (Speed Rate of the method).

To determine the SR of the method (i.e., the time to correctly identify the type of trapped cell/particle) we analyzed the minimum number of 2-seconds signal portions needed for a correct particle identification by the *i*LoF. A robust evaluation approach was also adopted to determine this parameter (Online Methods, Supplementary Fig. S7). According to the results, the *i*LoF method is characterized by a SR of 1.17 ± 0.51 2-seconds short-term signal portions, totaling 2.3 ± 1.0 seconds (Fig. 6(B)). However, in approximately 87% of the runs, the *i*LoF only needed a single signal portion for a 100% detection rate, which is a highly relevant performance attribute (Fig. 6(A)). Thus, despite only short-term signal portions being used for trapped cells/particles distinction, the set of 54 features chosen to describe them was significant enough to allow a correct classification using a single input test sample (Fig. 6 and Supplementary Fig. S8; Online Methods).

For more details about the performance of the *i*LoF in terms of the SR of detection, please see Supplementary Note S1.

### Data processing time reduction

To obtain performance results through the *Leave-One-Out* robust evaluation procedure, the *i*LoF had to be run for 770, 010, 000, 000 cycles (Fig. S9). Considering the computing characteristics of our machine, the initial duration for each one of the 29,250 evaluation runs was estimated to be of 339 seconds (Fig. S9, Online Methods). In total, all the computations required for the analysis to be completed would have been 339 × 29, 250 = 9915750*s* ≈ 115 days. Thus, we applied a multicore-based parallel computing approach to reduce the computation time corresponding to each one of the 29,250 evaluation runs by 42% (from 339 to 198 seconds), completing all the needed computations in 48 days. Further optimizations may be performed to additionally reduce the computation time for training model calculation. However, it is important to reinforce that after the calculation of the training model, the time needed for an unknown cell to be tested will only be 2 seconds.

## Discussion

In this study, we developed a novel, high-resolution, fast and portable method that we named “*i*LoF” (*intelligent* Lab on Fiber), which is able to trap and identify a single-cell. Its distinction power is transversal to highly similar cancer cells, which only differ in their corresponding surface glycosylation. This may constitute a major breakthrough in future detection methodologies of cancer and other diseases based on single cell fast screening. According to the results, our *i*LoF method has distinguished two gastric cancer cell models, whose differences were related to the length and complexity degree of cell surface glycans with accuracy values above 90%. This distinction ability is therefore aligned with a high-resolution detection technique. Recent evidence indicates that alterations in the glycosylation process are linked to tumor development^8^. Generally, shorter/truncated glycans at the surface of cancer cells are related with a poor prognosis in some cancer types, as previously described for sialyl Tn (STn)^34^. These phenomena are frequently associated with an incomplete glycans synthesis during cell glycosylation, in comparison with the cellular pathway under healthy conditions. To mimic these cellular alterations, we tested the *i*LoF method with two cell models derived from a gastric cancer cell line: the *HST6* and the *Mock* cells. The first was genetically modified in order to over-express an enzyme that causes a shift in the glycosylation pathway, leading to the expression of less complex and shorter glycans on their surface, such as STn. The *Mock* cells were transfected with the corresponding empty vector, displaying the characteristic glycosylation of the “wild-type”-parental cell model^19^. Aberrant glycosylation has been previously identified in various cancer related proteins, such as the glycoprotein CD44, which has been shown to be a major carrier of STn and associated to increased metastatic potential and poor survival in gastric cancer^9,35^.

By immobilizing the targets and further analyzing the light back-scattered signal arising from the trapped cell using an AI approach, the *i*LoF proves to be a highly robust alternative to the current methods for detecting different glycosylated cells^11^. Because the target remains immobilized (but untouched) during measurements, the acquired signal is not affected by cell movement-derived noise. Additionally, it does not require bulky equipment, fluorescent probes, antibodies or any type of functionalization, being mainly characterized by a microlens-like structure on the top of an optical fiber and a photodetector. Moreover, this microlens can be fabricated through a low-cost photopolymerization method. The *i*LoF ensured an average Accuracy, F-Measure and SR of 93%, 85% and 2.3 seconds, respectively, after a highly robust *Leave-One-Out*-based performance evaluation procedure. The AI algorithm was also trained to continuously verify if a cell/particle was trapped or not. These characteristics place this technology in a very competitive position in relation to the state-of-the-art methods^14,36^. Currently, the techniques able to detect alterations in post-translational modifications (e.g., cell glycosylation) are limited in number^10,14,36^. Given that these are slight cellular changes, only affinity and biochemical assays, involving fluorescence, or a highly sensitive spectral and imaging techniques are able to detect them^14,36^. However, the reliable profiling of cell glycans for clinical purposes through affinity assays requires external labels that are invasive, phototoxic, bleach when observed and has low spectral resolution^14^. Meanwhile, the mass spectrometry and Raman scattering have also been considered suitable for posttranslational cell modifications characterization^14,36^. However, these techniques are time-consuming, require dedicated instrumentation and a multi-wavelength scanning, being limited to the detection of molecules with specific vibrational states. Additionally, they do not allow the isolation of the analyzed cell for further purposes^14,36^, which is not the case for the *i*LoF where, after the identification procedure, the cell remains untouched and ready for further biological characterization. Nonetheless, the *i*LoF method has some limitations. Despite the capacity to detect a cancer cell from the universe of “known” entities for the classifier, the biological/physical/chemical mechanisms that allow the distinction of cancer cells with different glycosylation is not yet fully understood.

There are some possible explanations for the successful detection of such slight alterations. Because the cells differed on the type of glycans expressed at their surface, the most obvious explanation is related to the different interaction patterns of the light with the different “glycans coat” around each cell. The glycans might be arranged in a way that scatters more/less amount of light depending on the cell model, probably inducing interferences on the scattering signal, which are translated into different frequency components. The optical properties of each cell type (e.g., refractive index) could contribute to cell distinction through light scattering. However, to the best of our knowledge, this is the first time that this technique has been applied to distinguish cell glycosylation patterns in cancer. In this context, some fundamental information about cell optical properties is yet to be obtained - for example, cell refractive index distribution maps - to accurately explain the exact mechanism of distinction. In fact, the higher the refractive index difference between the target and the surrounding media, the higher the fraction of light that is scattered. The different spatial distribution of glycans - as already showed by mass spectrometry for other glycosylation moieties^36^ - over cell surface could increase the optical heterogeneity degree of each cell type. Additionally, the distribution of internal layers could be different in each cell model. The different layers could behave as resonant cavities when the light interacts to the cell, introducing phase changes into the scattering signal. This may be the reason why the phase parameters are among the 54 used features and present a high degree of contribution to the AI algorithm decision.

Thus, assuming that the slight dissimilarities between cell classes used in the experiment (with the same genetic background but different glycosylation) are reflected by tiny changes in refractive index, as already reported in previous studies about how glycans can change the optical properties of cells surface^37–39^, there is another reasonable explanation for the cell distinction mechanism behind the *i*LoF method. When the cell is under an optical trapping potential, a component of the gradient force can act as a harmonic optical restoring force which is counterbalanced by Brownian fluctuations^40,41^. According to the studies of O’Dell *et al*.^40^ and Lindner *et al*.^41^, the position of the cell under the influence of this trapping potential varies according to the following equation:

1 $${\sigma }^{2}(P(x,t))=\frac{{K}_{B}T}{{k}_{trap}}\left(\right.1-exp(\frac{-2{k}_{trap}Dt}{{K}_{B}T})\left)\right.,$$

where *σ*^2^ represents the variance, *P*(*x*, *t*) the probability of finding the cell in the position *x* at a time *t*, while *K*_*B*_, *T* and *D* represents the Boltzmann constant, the absolute temperature and the particle diffusion coefficient in the suspension media, respectively. The *k*_*trap*_ is a variable which is intrinsically correlated with the identity of the analyzed particle, being correlated with its refractive index and optical polarizability, but also with the gradient force that is exerted by the optical lens on it. Considering that the back-scattered signals collected from the trapped cells reflect the variability of cell position along time due to the “confined” Brownian motions around an equilibrium position (trapping position), the fluctuations found in the collected scattered patterns are intrinsically correlated with the optical properties of the trapped cell. However, the relation between these signal fluctuations and cell optical characteristics is only possible to study when a harmonic trapping potential is exerted on the analyzed microparticle. Based on these evidences, the *i*LoF use scattering signals collected by a cell under a trapping potential to classify its type since they reflect cell optical characteristics currently used as “optical fingerprints” for detecting specific molecules/proteins/biotargets attached or at the surface of biological particles^2,42,43^, as the refractive index or optical polarizability. Hereafter, we intend to conduct a detailed study to optically characterize each cell model for confirming this theory.

In conclusion, the developed methodology has the potential to be embedded in an affordable and easy-to-operate microchip that contains microfluidic channels to distinguish the presence of different models/subtypes of live cancer cells in circulating physiological fluids (e.g., blood, plasma, serum), while keeping the cells untouched for further biological characterization. It is also highly versatile, because it can be trained to distinguish completely novel targets, or introduce more classes to its range of detection. This novel method can therefore contribute to the development of the emerging field of personalized cancer medicine.

### Synthetic particles and cancer cells

Three types of solutions were prepared to test the proposed single-cell identification method. Two of them were composed by the differently glycosylated cancer cells (as described below) - *Mock* and *HST6* - suspended in PBS (Phosphate-Buffered Saline, 1x). The third solution contained eight *μ*m Polystyrene (PS) synthetic microspheres, which were also suspended in PBS (1x). These solutions were used to test the performance of our single-cell intelligent identification method based on 2D fiber trapping. PS particles were used as known control targets and to test the robustness of the *i*LoF identification performance considering complex/biologic versus simple/synthetic targets. Please see Supplementary Table S3 for more information about the solutions.

The human gastric cancer cell line MKN45 was obtained from the Japanese Collection of Research Bioresources (Tsukuba, Japan). Two different cell types were considered: *HST6* and *Mock* cancer cells. *HST6* cells are MKN45 cancer cells transfected with a vector over-expressing the ST6GalNAc1 glycosyltransferase, which is an enzyme leading to the biosynthesis of the tumor associated STn antigen (Neu5Ac*α*2-6GalNAc*α*-O-Ser/Thr). *Mock* refers to the control cells containing the empty vector^19^. Cells were cultured in RPMI 1640 GlutaMAX, HEPES medium (Gibco, Thermo Fisher Scientific, Waltham, MA, USA) supplemented with 10% heat-inactivated fetal bovine serum (Biowest, Riverside, MO, USA) and maintained at 37 ^°^C in an atmosphere of 5% CO_2_. Cultured cells were routinely tested for mycoplasma contamination and cell line identity was confirmed by STR profiling. For *i*LoF analysis, cells were detached from the flasks by gentle non-enzymatic cell dissociation (Gibco® Versene, ThermoFisher, Waltham, MA), resuspended in PBS and then plated into 35 mm *μ*-dishes (Ibidi, Germany).

The visual aspect of each cell model is provided in Fig. 2, which also includes the size statistical distribution information considering the population of cells analyzed in this study. Synthetic particles and cells were optically manipulated during experiments under controlled temperature, atmosphere and humidity, at 37 ^°^*C* and 5% CO_2_.

### Glycan characterization of the cancer cells

The phenotypic glycan alteration induced by the overexpression of ST6GALNAC1 enzyme was analysed by flow cytometry. Cells were detached using non-enzymatic cell dissociation solution (Gibco® Versene) and stained with previously complexed anti-STn monoclonal antibody (clone TKH2^44^) with anti-mouse IgG Alexa Fluor®-488-conjugated secondary antibody for 20 minutes at 4 ^°^C. Cells were strained, labeled with propidium iodide and measured using BD FACSCanto^TM^ II (BD Biosciences, San Jose, CA). Two independent experiments were conducted. Data were analyzed using FlowJo (BD Biosciences, San Jose, CA).

Further glycomic analyses were performed to characterize the cancer cell glycosylation by Liquid Chromatography/Electrospray Ionization Tandem Mass Spectrometry (LC-ESI-MS/MS). Samples were prepared and analyzed as described in^45^. Briefly, frozen cell pellets (10^7^ cells) of *HST6* transfected cells or *Mock* were directly resuspended in 7 M urea, 2 M thiourea, 40 mM Tris, 2% CHAPS, 10 mM DTT and 1% protease inhibitor (Sigma-Aldrich, St. Louis, MO). The cell membranes were disrupted by sonication and the viscosity of the lysates was reduced by benzonase® nuclease (250 units, Sigma-Aldrich). Iodoacetamide was added and supernatants collected. *N*-linked oligosaccharides of the supernatant glycoproteins were released on 10 kDa cut-off spin-filter (PALL, Port Washington, NY) by PNGase F (Prozyme, Hayward, CA). The released *N*-glycans were collected, dried in Speedvac and reduced overnight with 0.5 M *NaBH*_4_, 10 mM *NaOH*. *O*-linked oligosaccharides were released from retained glycoproteins in spin-filter by reductive *β*-elimination (0.5 M *NaBH*_4_, 50 mM *NaOH*). Reactions were quenched with 1 *μ*l of glacial acetic acid and *N*- and *O*-glycans were desalted and dried. Released glycans were analyzed by LC-ESI-MS/MS using a column containing 5 *μ*m porous graphitized carbon (PGC) particles (Thermo Scientific, Waltham, MA). Glycans were eluted using an acetonitrile gradient in 10 mM *NH*_4_*HCO*_3_. The eluted *N*- and *O*-glycans were detected using a LTQ ion trap mass spectrometer (Thermo Scientific) in negative-ion mode of electrospray ionization. The data were processed using the *Xcalibur* software (version 2.0.7, Thermo Scientific) and manually interpreted from their MS/MS spectra. The identified glycan structures were quantified by the area under the peak at the extracted ion chromatogram.

### Fabrication of the polymeric microlens

The polymeric lens used in this study was fabricated through a guided wave photo-polymerization method developed by Soppera *et al*.^21^ in collaboration with our lab^4,15^. It has already shown to be suitable to trap synthetic microparticles and non-human cells^4,15^. Its fabrication method is based on the assemble of cross-linked polymeric structures through monomers linking triggered by light^4,21^. The monomer and photo-initiator used in this reaction was the pentaerythriol triacrylate (PETIA) and the Bis(2,4,6-trimethylbenzoyl)-phenylphosphineoxide (which is commercially known as Irgacure 819), respectively. Considering the properties of the photo-initiator, a violet diode 405 *nm* laser (LuxX cw, 60 *mW*, Omicron) was used to trigger the cross-linking reaction. At first, an optical fiber (*Thorlabs SM 980-5.8-125*) was cleaved at one of its extremities and was positioned vertically in a moving stage, while the laser was aligned to be injected in its distal end to excite the fundamental mode. The cleaved optical fiber extremity was dipped into a solution containing 0.2% in weight of Irgacure 819 (relatively to the monomer). After being removed from this solution, the polymer drop formed in the fiber extremity was cured by a laser power of at least 5 *μ*W at 405 *nm*, during 60 seconds. Then, the remaining liquid was washed out from the polymer tip using ethanol. During polymer solidification, the increase on the refractive index of the growing structure generates a self-guiding effect. The visual aspect of the spherical-lensed tip obtained through this process is provided in Fig. 1(A).

### Optical trapping and back-scattered signal acquisition setup

The experimental setup used for manipulating particles/cells and acquiring the back-scattered signal consisted of an inverted microscope (Zeiss Axiovert 200M, *Carl Zeiss*®) connected to a computer with the control software *Micro-Manager 1.3* (http://www.micro-manager.org) installed and equipped with a digital camera (CoolSnap HQ, *Roper Scientific*); a motorized micromanipulator (InjectMan®, Eppendorf®) with three degrees of freedom (*x*, *y*, *z* and angular); a photodetector (PDA 36A-EC, *Thorlabs*); a 980 *nm* laser (500 mW, *Lumix*, ref. *LU0980M500*) and a data acquisition board (DAQ, from *National Instruments*) - please see the scheme of Fig. 1(B). An optical fiber coupler (configuration type 1 × 2, 50/50@980 *nm*) was used to connect the 980 *nm* laser and the photodetector for back-scattered signal acquisition. The fabricated optical fiber tip was then spliced to the output of the optical fiber coupler and inserted into a metallic capillary that was positioned onto the motorized manipulator and tilted at 50^°^, because trapping effects are only possible at inclination angles >30^°^^4^. This bi-directional configuration allowed the light to be guided through the fiber and, at the same time, the the laser light back-scattered signal was collected by the photodetector. The latter was connected to one of the analog-to-digital output ports of the DAQ for signal transmission and recording at a laptop using the Data Acquisition Toolbox from MATLAB 2015a®. The trapping laser light of 980 *nm* was modulated by a sinusoidal signal (fundamental frequency of 1 KHz) digitally generated at a sampling rate of 5 KHz using a custom-build MATLAB script and externally injected in the laser driver through one of the digital-to-analog ports of the data acquisition board. This modulation reduced the interference of the 50 Hz local electrical grid component and other noisy components of the signal. The laser input signal modulation has already been shown to be an important procedure in this type of experiment^5,15,46,47^. The output laser diode power was set to ≈120 mW at the output of the 50/50@980 fiber coupler single entry during the experiment, to ensure a stable targets trapping/immobilization. This value was determined in accordance with the values used in the literature for optical delivery, collection and manipulation effects through optical fibers considering the selected wavelength value range^48^, and to cause as little damage as possible to the cells.

### Experimental trapping force calculation

The trapping forces exerted by the proposed lenses on each target type were calculated through the *Drag Force* method, which is based on a revised form of the Stokes equation and considers that the trapped particle is close to a boundary (in this case, to the bottom of the Ibidi® dish)^4,49^. According to the *Drag Force* method, the total trapping force exerted on the target results from the sum of the inertial and drag forces^49^:

2 $${F}_{T}={F}_{inertial}+{F}_{drag}=m\frac{{\partial }^{2}s}{\partial {t}^{2}}+6\pi \xi \eta r\frac{\partial s}{\partial t},$$

where *m* is the mass of the manipulated particle, *s*(*t*) represents the target trajectory during manipulation, *ξ* a correction factor for the proximity of the particle to the trapping chamber (*ξ* = 3.08)^4^, *η* the viscosity of the media (in this particular case, PBS, *η* = 1 × 10^−3^*Pa*^50^) and *r* represents the radius of the particle. However, the Reynolds number associated with this particular scenario is very low, and the inertial force can be considered negligible^49^. Thus, the optical trapping force can be calculated by determining the drag force^49^, acting on the particle:

3 $${F}_{T}=6\pi \xi \eta r\frac{\partial s}{\partial t}.$$

To obtain the velocity of the particle $(\frac{\partial s}{\partial t})$ due to optical trapping, the following assay was performed for each type of particle (two cancer cells from each type and one PS particle), while the target trajectory was recorded using the digital camera at a frame rate of 4 Hz. After each particle was stably trapped in front of the lensed tip, as depicted in Supplementary Fig. S2, the laser was turned off and the fiber tip was moved a few micrometers away from the target towards the −*x* direction (towards the left). Then, the laser was turned on. The particle was consequently attracted to the equilibrium position (trapping position), while its trajectory was recorded. This procedure was repeated by displacing the fiber tip towards the +*x* direction. Each displacement was recorded for three times for each direction to obtain a statistical profile of the trapping forces. After video acquisition, the particles’ trajectories were tracked using the *CellTracker*^51^ MATLAB®-compatible software. Then, the particles’ position for each time point was fitted to the Langevin approximation^52^ and the particles velocity during the restoring movement was calculated for each type of target, transversal movement direction (−*x*, +*x*) and repetition. The trapping forces profile was then traced for each direction, based on the equation defined in 3, and then compared between different cell/particle type.

### Back-scattered signal acquisition and processing

After the optical setup was correctly mounted and turned on, a simple assay was carried out for each one of the solutions described in Supplementary Table S3, to solve the following four classes problem using back-scattered signal derived features: “Class 1: No particle trapped”; “Class 2: *Mock* cancer cell trapped”; “Class 3: *HST6* cancer cell trapped” and “Class 4: Polystyrene microparticle trapped”. A drop of each solution was placed over a 35 mm Ibidi® micro rounded dish mounted in the inverted microscope. Then, the polymeric lensed optical fiber tip was immersed into this sample, with the help of the microscope imaging system. After the polymeric lens had been carefully positioned in front of an isolated cell/particle, the laser was turned on and, once the target was immobilized due to optical trapping (as depicted in Supplementary Fig. S2, the back-scattered signal was acquired. Similarly to the procedure adopted in a previous experiment also conducted by our lab^15^, 80 seconds (80 s) of back-scattered signal were acquired per cell/particle through a photodetector (PDA 36A-EC, *Thorlabs*) connected to an analog-to-digital converter of the data acquisition board (*National Instruments* DAQ) at a sampling rate of 5 kHz. Signal acquisitions for the case of no particle in front of the tip were also conducted, to represent the class "No particle trapped”. The inclusion of this class in the proposed Supervised Learning problem could be relevant to find the best set of training parameters to continuously verify if a given particle was trapped or not. These acquisitions were performed by moving the polymeric tip into an empty area, where, although the laser remained turned on, no particle was trapped. Several “No particle trapped” acquisitions were performed to increase the samples’ variability and to then evaluate the robustness of the proposed method by considering different acquisition spots into the same solution. Then, the acquired signals (considering all the classes) were processed according to the scheme of Fig. 5. A MATLAB 2015a® custom-built script was used for both signal acquisition and processing. Signal Processing and Statistics toolboxes from MATLAB® were used for signal processing and in subsequent analysis steps. A total of 4,240 seconds of back-scattered signal was acquired, considering all of the classes.

After each acquisition, the original signal was passed through some processing steps. After signal processing, the obtained dataset was composed of back-scattered signal portions of 2 seconds (representing each sample of the dataset). After removing the noisy 2 seconds portions in the artifact rejection stage, a set of 54 features characterizing each 2 seconds signal portion was created - see Supplementary Table S4 for a description of the final dataset obtained. Then, the Random Forests^32^, a very effective classifier in solving complex problems which involve non-linearly separable classes, was applied to identify the type of particle trapped. A scheme summarizing all the steps conducted during signal processing and classification is depicted in Supplementary Fig. S3.

#### Signal processing steps

A custom-built MATLAB® 2015a script that requires functions from both the Signal Processing® and Statistics® toolboxes was created for signal processing. After acquisition, the signal was at first filtered using a second-order 500 Hz Butterworth high-pass filter, because this type of filter was already successfully used to statistically differentiate synthetic and simple biological cells in previous studies conducted by our laboratory^15,46^. Considering that laser trapping signal was modulated with an external 1 kHz sinusoidal signal, this type of filter would remove noisy low-frequency components of the original signal, such as the 50 Hz electrical grid component. Then, each whole 80-seconds acquisition (400 k samples) was split into short-term signal epochs of 2 seconds (10 k samples). Independently of the type of features used in this kind of problems, it is important that their raw signals have the highest possible signal-to-noise ratio (SNR)^31^. Thus, the *z-score* of each 2-seconds signal portion was computed to exclude noisy short-term portions whose value exceeded the threshold of ∣*z* − *score*∣ > 5^31^. Sketches of processed signal portions for each type of cell/particle trapped are provided in Fig. 5. After signal processing, 54 features based on time and frequency domain of each 2-seconds back-scattered short-term signal portion were computed.

### Artificial intelligent-based cells/particles classification method

According to the proposed method, cell classification is possible by training an Artificial Intelligence Supervised Machine Learning algorithm, which will be able to automatically classify novel instances (novel particles). However, at first, a set of 54 features characterizing each 2-seconds short-term back-scattered signal must be calculated to provide to the learning algorithm with the information that it needs to distinguish between differently glycosylated tumoral cells.

#### Features

The capacity of 43 of the 54 features set used in this classification problem to distinguish different particles was already assessed in a previous study^15^. These features were created considering several attributes already used in similar differentiation problems, such as macro-targets type identification through scattering signal acquired using photodetectors or other kind of “event counter” equipment, including underwater fish species recognition or object identification in the surrounding environment (in air, water, etc.)^53^. To the best of our knowledge, this type of feature has never been used in micron-sized targets such as cells. This feature set can be divided into two main types: time- and frequency-domain^15^. The first type can be also subdivided in time-domain statistics attributes and time-domain histogram-derived parameters. The frequency-domain features can be also grouped into Discrete Cosine Transform (DCT)-derived type and Wavelet-derived features^15^. All of the 54 features used in the proposed method can be found in Table 1.

The following time-domain statistics features were extracted from each 2-seconds signal portion: Standard Deviation (SD), Root Mean Square (RMS), Skewness (Skew), Kurtosis (Kurt), Interquartile Range (IQR), Entropy (E). Considering that the Nakagami distribution have been widely used to describe the back-scattered echo in statistical terms^54^, mainly within the biomedical area, the Probability Density Function (PDF)-derived *μ*_*Nakagami*_ and *ω*_*Nakagami*_ parameters that better fit the approximation of each 2-seconds signal portion distribution to the Nakagami distribution were also considered^15^.

Considering the ability to capture minimal periodicities of the analyzed signal, the associated coefficients are uncorrelated and due to the fact that, in contrast to the Fast Fourier Transform (FFT), it does not inject high-frequency artifacts in the transformed data, the Discrete Cosine Transform (DCT)^55^ was applied to the original short-term signal portions to extract frequency-derived information. Considering that the first *n* coefficients of the DCT of the scattering echo signal are defined by the following equation^53^:

4 $${E}^{DCT}[l]=\mathop{\sum }\limits_{k=0}^{N-1}\varepsilon [k]\cos \left[\frac{\pi l(2k+1)}{2N}\right],\quad for\quad l=1...n,$$

in which *ε* is the signal envelope estimated using the Hilbert transform; by sorting the DCT coefficients from the highest to the lowest value of magnitude and obtaining the following vector:

5 $$y=\left(\right.{E}^{DCT},...,{E}^{DCT}[{l}^{n}]{\left)\right.}^{T},$$

in which *E*^*DCT*^[*l*^1^] represents the highest DCT coefficient in magnitude, it is possible to determine the percentage of the total amount of the signal energy that each set of coefficients represents (organized from the highest to the lowest one). Each percentage value regarding each coefficients set (from the first to the n^*th*^ coefficient) can be obtained by dividing the norm of the vector formed by the first till the *n*^*th*^ coefficient by the norm of the vector composed by all the *n* coefficients. Thus, the following DCT-derived features were used to characterize each 2 s signal portion: the number of coefficients needed to represent 98% of the total energy of the original signal (*N*_*DCT*_), the first 20 DCT coefficients extracted from the vector defined in 5, the Area Under the Curve (AUC) of the DCT spectrum (from 0 to 2.5 kHz) (*AUC*_*DCT*_), the maximum amplitude of the DCT spectrum (*Peak*_*DCT*_) and the signal power spectrum obtained through the DCT considering all the values within the frequency range analyzed (*P*_*DCT*_) - please see Table 1.

The remaining 12 features were extracted after 2-seconds signal portion decomposition using wavelets^56^ (see Table 1). Two mother wavelets - *Haar* and Daubechies (*Db10*) - were selected to characterize each back-scattered signal portion. These two types were chosen due to their simplicity and considering the fact that they were already successfully used to decompose back-scattered signals in underwater scenarios for macro-objects recognition^53^. Six features for each type of mother Wavelet based on the relative power of the Wavelet packet-derived reconstructed signal (one to six levels) were therefore extracted from each short-term 2-seconds signal^15^.

#### Cancer cells/particles automatic classification using AI

The AI classification algorithm chosen for this problem was the Random Forests^32^. The concept behind Random Forests consists in growing an ensemble of Decision Trees and then letting them vote for the most popular class^32^. They have been successfully applied to a myriad of Biomedical problems, because they are very effective in distinction problems involving non-linearly separable classes and more robust to overfitting effects in comparison with equivalent classifiers in terms of performance^32^. However, to attain the best performance, there are three important parameters that must be optimized before applying Random Forests: its number of decision trees (the corresponding number of generated trees will therefore vote for the most popular class); the number of predictors to sample, which represents the number of features to select at random for each decision split; and the minimum leaf size (minimum number of samples per tree leaf)^32^. Usually, the most suitable number of predictors to sample corresponds to the square root of the number of features used in classification^32^. However, the most adequate combination of values for these parameters should be tuned into the classifier training stage^32^. The parameter set that was tuned and corresponding range values for optimizing the classifier can be found in Supplementary Table S5.

To avoid overfitting, the classifier must be tested using new samples, which were never involved in the classifier training phase. Additionally, the samples used in the test must belong to a subject or entity whose samples were never presented to the classifier during the training^33^. Usually, the *Leave-One-Out* procedure^33^ is used to ensure that the data used for evaluating the performance of a classifier belong to a subject/entity that had never been involved in the training. According to this validation method, if a dataset is composed by data from *n* subjects/entities, then the test set must be divided accordingly in *n* testing rounds. Then, in each round, the data from a subject/entity is used to test and the data from the remaining *n* − 1 subjects/entities used for classifier training. Subsequently, in the following round, the data subset from another subject/entity that was selected in the previous round for classifier training is used separately to test the classifier. Then, the classifier performance is determined based on the mean values obtained after the *n* testing rounds. We adopted a similar scheme to validate our method, where each cell/particle was considered an entity/subject^31,33^. Thus, we conducted *n* = 13 × 15 × 15 × 10 = 29, 250 evaluation runs. Each test set was composed of the attributes set corresponding to four particles, each belonging to one of the four classes considered, while the remaining 53 − 4 = 49 particles were assigned to the training set, for each one of the 29,250 evaluation runs. By considering all of the possible combinations of particles between training and test sets, all of the considered particles were used in the training as in the test set ensuring, at the same time, that the data used in the test set were never involved in the training phase for each *n*^*th*^ evaluation run^31,33^. Thus, the robustness of the method could be evaluated, while avoiding the kind of situations where a classifier is very well rated or the records in testing data are very hard to classify because one or more of the entities involved in its validation were exclusively included in the test or in the training phase. A scheme explaining both training and testing procedures can be found in Supplementary Fig. S4(A).

The function *TreeBagger* from Statistics Toolbox from MATLAB® was used to generate the Random Forests for classification. The 54 features from Table 1 were used to characterize each 2 s signal sample. During the training phase, the most suitable combination of values between the three parameters “number of trees”, “number of predictors to sample” and “minimum leaf size” - please see Supplementary Table S5 - was determined, based on the higher average F-Measure value attained using the five-folds *Cross-Validation* method, for each *n* evaluation run. However, due to intrinsic amplitude differences between features both at the intra- and inter-particles/cells level, a normalization procedure was applied to each sample of the dataset, for each evaluation run. Training samples mean value across each feature was subtracted to each data sample from that feature, and then divided by the corresponding feature standard deviation^31^. Test input samples were normalized also according to this procedure, using the previously obtained training mean and standard deviation for each feature. This allowed us to map the novel test features vectors in the training features space - see stage (7) from scheme of Supplementary Fig. S3. The performance of the proposed method was evaluated considering the mean test Accuracy and F-Measure across the 29,250 evaluation runs.

### Determining the speed Rate (SR) of the *i*LoF method

Apart from analyzing the algorithm performance by taking into account a given number of signal portions in the test set (corresponding to the whole acquisition period for each particle), the minimum number of signal portions needed for a correct identification by the algorithm was also evaluated to determine the corresponding SR. This required the average number of signal portions that were used to be determined until the algorithm could correctly identify each particle with 500 repetitions. This value is commonly used in the literature^57^. This procedure was therefore repeated for each one of the 29,250 evaluation runs, where each run represented a different combination between particles chosen for training and testing the classifier. After each evaluation run (i.e., by using one of the *n* combinations), the classification algorithm output label was evaluated for each selected particle/cell, while taking into account only one signal portion chosen randomly from the corresponding test set. If the output label did not correspond to the ground truth, then another signal portion sample that had not been chosen yet was randomly selected from the set of back-scattered signal portions, until the classification algorithm correctly identified the current particle, or until all the signal portions in the test set had been used. This procedure was performed 500 times. A description scheme of this algorithm performance evaluation procedure is provided in Supplementary Fig. S7.

The SR of the method was obtained by taking into account the average value of the number of signal portions needed to identify each particle across 500 repetitions and along the 29,250 evaluation runs. SR was obtained for each evaluation run by training the algorithm using the parameters for which the most accurate *Cross-Validation* was previously determined.

### Considerations on reducing the data processing time

Because we intended to evaluate the robustness of the method by training and testing it for all the possible combinations between the evaluated particles (*n* = 29, 250 different combinations), this led to a highly time-consuming computational problem. For each *n*^*th*^ evaluation run, the five-fold *Cross-Validation* method had to be conducted, in a first stage, for each one of the combinations between the three training parameters (“number of trees”, “number of predictors to sample” and “minimum leaf size”), totaling 180 different parameter combinations, to determine the most suitable cross-validated training parameters set. Thus, considering that we chose a five-fold scheme to tune the training parameters, a Random Forests classifier had to be trained and tested during the *Cross-Validation* stage for 5 × 180 = 900 times. Additionally, and taking into account that we had to conduct *Cross-Validation* for each *n* different combinations between particles, the algorithm was trained and tested within the *Cross-Validation* stage for 29, 250 × 900 = 26, 325, 000 times, in total.

After determining the best cross-validated training parameters, we had to train the algorithm using these settings for each of 29,250 different combinations between particles. Then, each trained classifier for each one of the 29,250 different combinations was tested to obtain the corresponding accuracy, F-Measure and SR performance values. In summary, starting with *Cross-Validation*, passing through training and ending with testing phase, the algorithm had to be run for 29, 250 × 900 × 29, 250 = 770, 010, 000, 000 times. Considering the associated time consuming computation, we use a multicore-based parallel computing approach to solve the problem. Considering each evaluation run from the 29,250 that had to be performed, we distributed the five iterations relative to the five-fold *Cross-Validation* problem for the eight cores of our machine (an iMac 2017 from Apple Inc. with 4.2 GHz Intel Core *i7* processor, a 64 GB 2400 MHz DDR4 memory and eight cores), by taking part of the functionalities of the Parallel Computing Toolbox from MATLAB® (Supplementary Fig. S9).

### Statistical analysis

Statistical tests were performed to confirm whether the cell diameters differed among the differently glycosylated cancer cell models. We also applied statistics to investigate whether the transversal gradient force magnitude exerted by the fabricated microlens-like structure on each cell was significantly different. At first, we applied the Shapiro-Wilk Normality test^58^ to verify if each variable involved in the analysis followed a normal distribution. Both the number of samples and significance of each test applied (*P* < 0.05 for significant differences) are provided in the figure legend or the results section of the main text. Because all of the analyzed variables were normally distributed (*P* > 0.05; *Shapiro-Wilk Normality Test, two-tailed*), parametric statistical tests were considered. The Student’s T-test for independent samples (two tailed) was applied for comparing the two measures (cell diameter and trapping force magnitude). A correction for multiple comparisons was introduced when trapping force magnitude along cell position relatively to the trapping point was compared between cells. The *Bonferroni* criteria^59^ was considered to correct the obtained p-value for multiple comparisons. Statistical tests were performed using the Statistics Toolbox for MATLAB®.

## Supplementary information

Video S1.

Supplementary Material.
