# Author Correction: *i*LoF: An intelligent Lab on Fiber Approach for Human Cancer Single-Cell Type Identification

**DOI:** 10.1038/s41598-020-67916-4

**Published:** 2020-06-26

**Authors:** Joana S. Paiva, Pedro A. S. Jorge, Rita S. R. Ribeiro, Meritxell Balmaña, Diana Campos, Stefan Mereiter, Chunsheng Jin, Niclas G. Karlsson, Paula Sampaio, Celso A. Reis, João P. S. Cunha

**Affiliations:** 1 INESC TEC - INESC Technology and Science, Porto, Portugal; 2 Physics and Astronomy Department, Faculty of Sciences, University of Porto, Porto, Portugal; 3 Faculty of Engineering, University of Porto, Porto, Portugal; 4 i3s - Instituto de Investigação e Inovação em Saúde, Universidade do Porto, Porto, Portugal; 5 IPATIMUP - Institute of Molecular Pathology and Immunology, University of Porto, Porto, Portugal; 6 Department of Medical Biochemistry and Cell Biology, Institute of Biomedicine, Sahlgrenska Academy, University of Gothenburg, Gothenburg, Sweden; 7 IBMC - Instituto de Biologia Molecular e Celular, Universidade do Porto, Porto, Portugal; 8 Instituto de Ciências Biomédicas Abel Salazar, University of Porto, Porto, Portugal; 9 Faculty of Medicine, University of Porto, Porto, Portugal; 10 Present Address: 4DCell, Paris, France; 11 Present Address: IMBA, Institute of Molecular Biotechnology of the Austrian Academy of Sciences,, Vienna BioCenter Campus, 1030 Vienna, Austria

Correction to: *Scientific Reports* 10.1038/s41598-020-59661-5, published online 21 February 2020

The Acknowledgements section in this Article is incomplete.

“This work was partially funded by the projects NanoSTIMA and NORTE-01-0145-FEDER-000029, both supported by the North Portugal Regional Operational Program (NORTE 2020), under the PORTUGAL 2020 Partnership Agreement, and through the European Regional Development Fund (ERDF); and by the Portuguese Foundation for Science and Technology, within the scope of the PhD grant PD/BD/135023/2017 and the projects: PTDC/BBB-EBI/0567/2014 (to CAR) and UID/BIM/04293/2013. It was also funded by FEDER funds through the Operational Programme for Competitiveness Factors-COMPETE (POCI-01-0145-FEDER-016585; POCI-01-0145-FEDER-007274; PPBI-POCI-01-0145-FEDER-022122). MB acknowledges the Marie Sklodowska-Curie grant agreement No. 748880.”

should read:

“This work is financed by National Funds through the Portuguese funding agency, FCT - Fundação para a Ciência e a Tecnologia, within project UIDB/50014/2020. It was also partially funded by the projects NanoSTIMA and NORTE-01-0145-FEDER-000029, both supported by the North Portugal Regional Operational Program (NORTE 2020), under the PORTUGAL 2020 Partnership Agreement, and through the European Regional Development Fund (ERDF); and by the Portuguese Foundation for Science and Technology, within the scope of the PhD grant PD/BD/135023/2017 and the projects: PTDC/BBB-EBI/0567/2014 (to CAR) and UID/BIM/04293/2013. It was also funded by FEDER funds through the Operational Programme for Competitiveness Factors-COMPETE (POCI-01-0145-FEDER-016585; POCI- 01-0145-FEDER-007274; PPBI-POCI-01-0145-FEDER-022122). MB acknowledges the Marie Sklodowska-Curie grant agreement No. 748880.”

Additionally, there is an error in the first column of Table 1, where

“Frequency domai”

should read:

“Frequency domain”

