# Supplementary material for: iLoF: An intelligent Lab on Fiber Approach for Human Cancer Single-Cell Type Identification
Source: Sci Rep. 2020 Feb 21;10:3171. doi: 10.1038/s41598-020-59661-5 (PMC7035380; doi:10.1038/s41598-020-59661-5)
Supplement: Supplementary file 2 — Supplementary Material. [file 41598_2020_59661_MOESM2_ESM.pdf]

*i*LoF: An intelligent Lab on Fiber Approach for Human Cancer Single-Cell Type Identification

Joana S. Paiva[1,2,3], Pedro A. S. Jorge[1,2], Rita S. R. Ribeiro[1,†], Meritxell Balmaña[4,5,††], Diana Campos[4,5], Stefan Mereiter[4,5,††], Chunsheng Jin[6], Niclas G. Karlsson[6], Paula Sampaio[4,7], Celso A. Reis[4,5,8,9], João P. S. Cunha[1,3,*]

[1] *INESC TEC - INESC Technology and Science, Porto, Portugal*

[2] *Physics and Astronomy Department, Faculty of Sciences, University of Porto, Portugal*

[3] *Faculty of Engineering, University of Porto, Porto, Portugal*

[4] *i3s - Instituto de Investigação e Inovação em Saúde, Universidade do Porto, Portugal*

[5] *IPATIMUP - Institute of Molecular Pathology and Immunology, University of Porto, Porto, Portugal*

[6] *Department of Medical Biochemistry and Cell Biology, Institute of Biomedicine, Sahlgrenska Academy, University of Gothenburg, Gothenburg, Sweden*

[7] *IBMC - Instituto de Biologia Molecular e Celular, Universidade do Porto, Portugal*

[8] *Instituto de Ciências Biomédicas Abel Salazar, University of Porto, Porto, Portugal*

[9] *Faculty of Medicine of the University of Porto, Porto, Portugal*

[†] *Rita S. R. Ribeiro is currently with 4DCell, Paris, France.*

[††] *Meritxell Balmaña and Stefan Mereiter's present address is IMBA, Institute of Molecular Biotechnology of the Austrian Academy of Sciences, Vienna BioCenter Campus, 1030 Vienna, Austria*

Supplementary Material

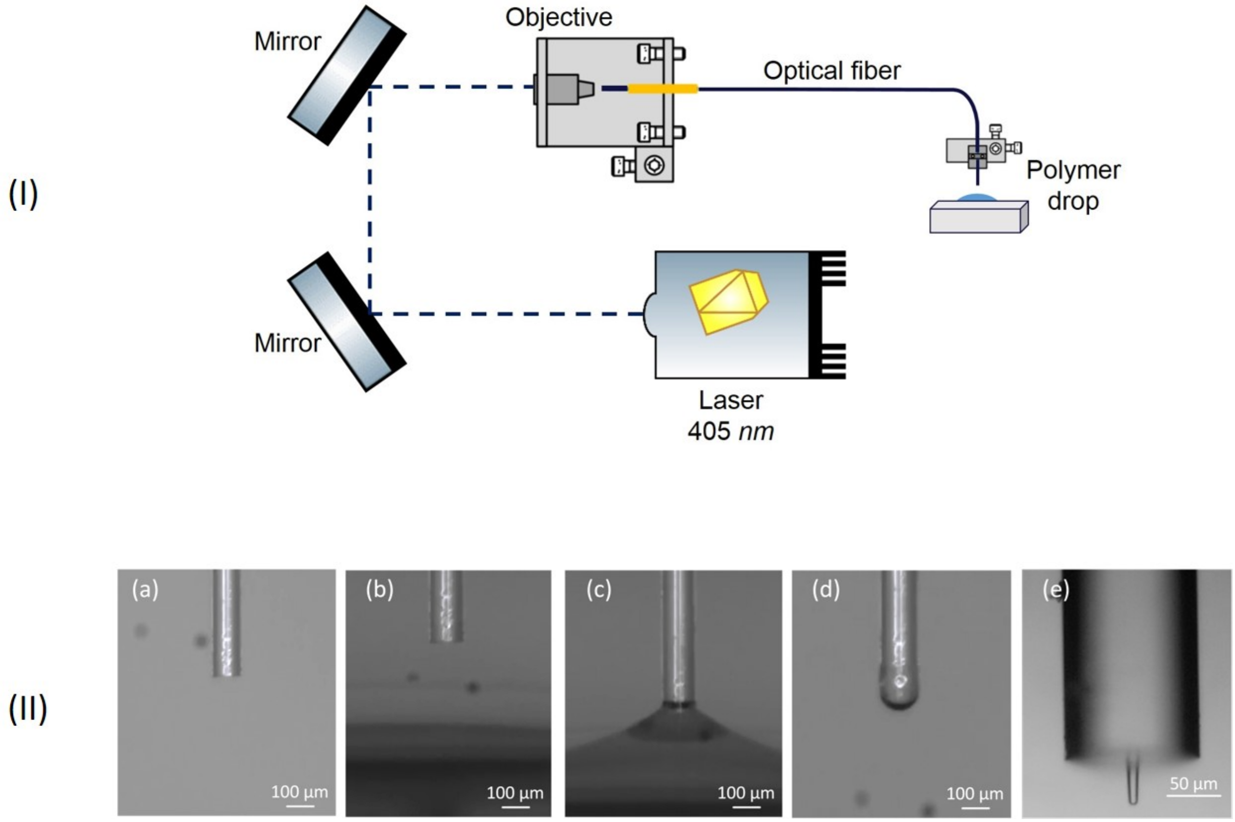

Figure S1: Photo-polymerization fabrication method of microlenses on the top of optical fibers [1]. (I) Scheme of the optical setup used to couple the 405 *nm* laser used to polymerize the micro structures on the optical fiber tips. (II) Fabrication process stages: (a) At first, a single mode (SM) optical fiber was cleaved at one of its extremities and (b) was positioned vertically in a moving stage. (c) Then, the cleaved optical fiber extremity was dipped into a solution containing the monomer and photo-initiator. (d) After being removed from this solution, the polymer drop formed in the fiber extremity was cured by turning on the laser. (e) Then, the remaining liquid was washed out from the polymer tip using ethanol and it was possible to obtain a waveguide-like structure on the top of the optical fiber with the aspect depicted in the figure.

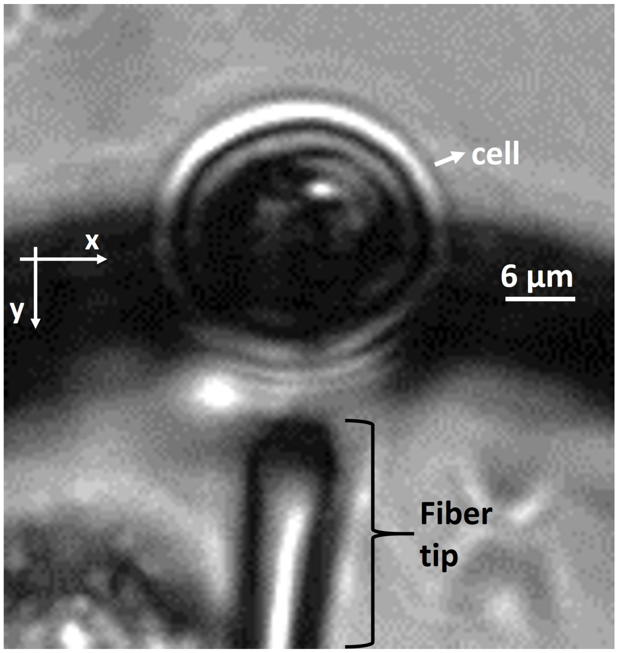

Figure S2: A *HST6* cancer cell stably immobilized in front of the optical fiber tip due to optical trapping.

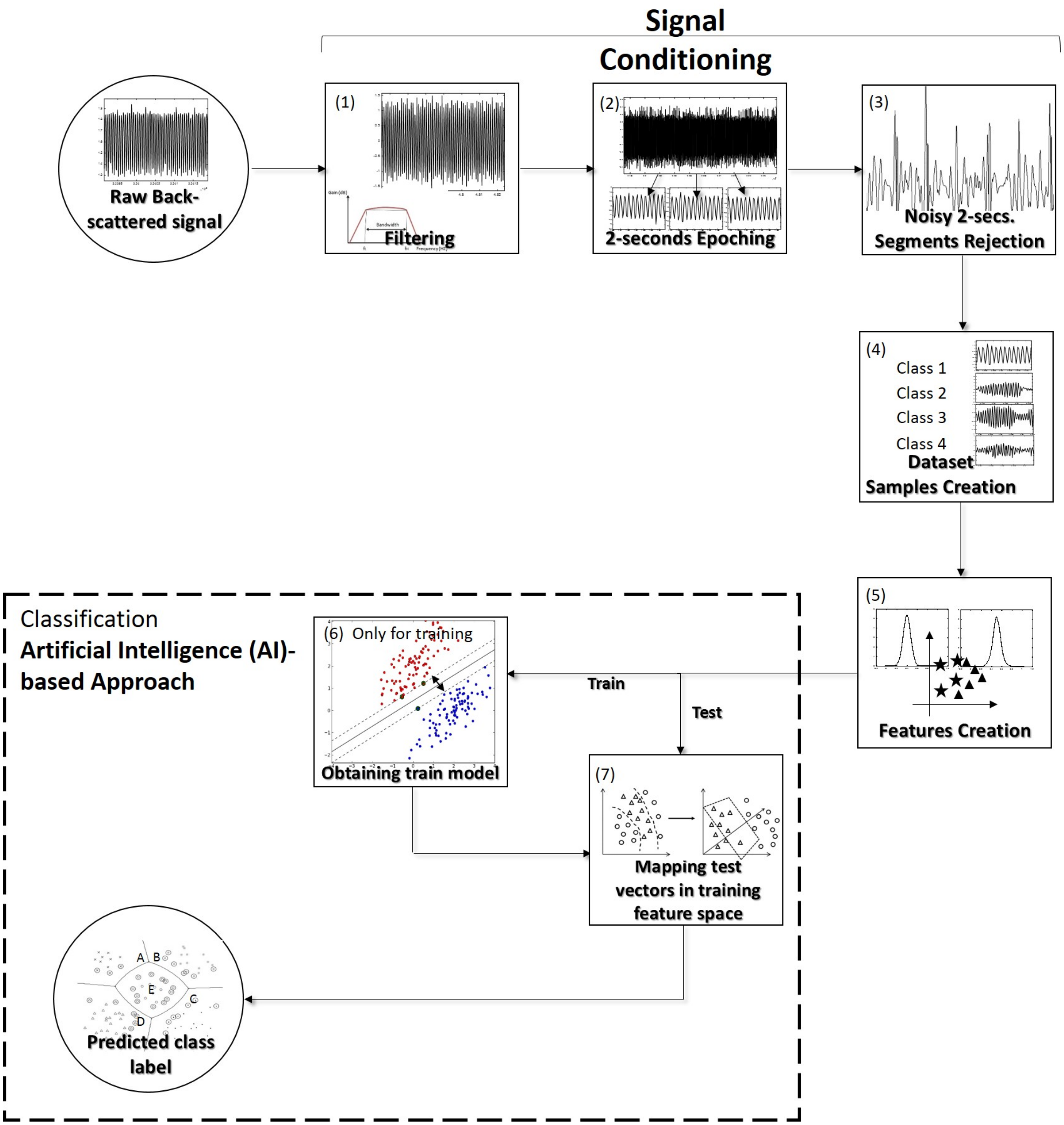
Signal Conditioning
Raw Back-scattered signal
(1)
Filtering
(2)
2-seconds Epoching
(3)
Noisy 2-secs. Segments Rejection
(4)
Class 1
Class 2
Class 3
Class 4
Dataset Samples Creation
(5)
Features Creation
Train
Test
Classification
Artificial Intelligence (AI)-based Approach
(6) Only for training
Obtaining train model
(7)
Mapping test vectors in training feature space
Predicted class label
A
B
C
D
E

Figure S3 *(preceding page)*: Scheme explaining all the back-scattered signal processing, analysis and cells classification steps. (1) At first, each whole acquisition was filtered using a second-order 500 Hz Butterworth high-pass filter. (2) Then, each 80-seconds entire acquisition from each class and particle/cell was split into 2-seconds signal epochs. (3) Each signal portion was z-scored and discarded if one of its values exceeded $|zscore|$ >5, during artifact rejection. (4) After these steps, it was possible to obtain a dataset with 2 seconds signal portions with a reasonable Signal-to-Noise Ratio (SNR) for the particle/cell classification to be possible. (5) Then, the 54 signal-derived features set used for classifying each cell according to the four classes ("Class 1: No particle"; "Class 2: *Mock* tumor cell"; "Class 3: *HST6* tumor cell"; "Class 4: Polystyrene microparticle") was generated. (6) In the particle classification, a Supervised Learning classifier will be trained using the 54 signal-derived features and its settings optimized to the selected problem. (7) Then, the most suitable model previously obtained in the train will be used to map input test attributes belonging to novel cells into the training feature space, to obtain the predicted label (to which class the cell/particle under test belongs).

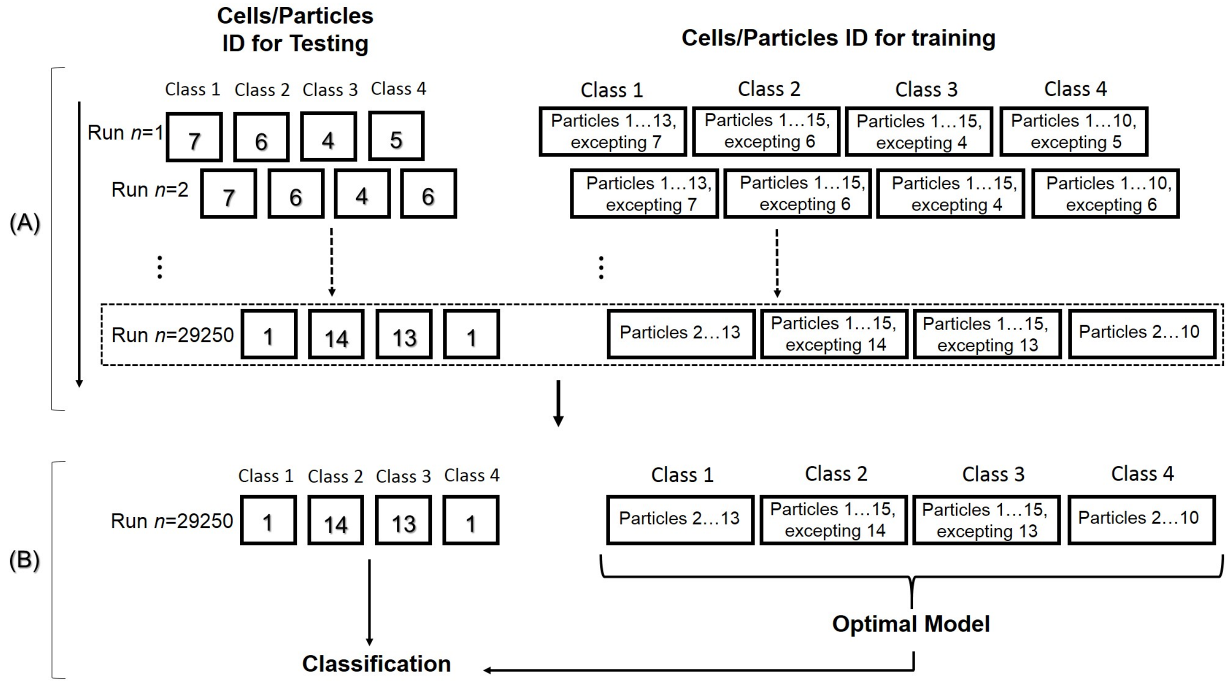

Figure S4: Scheme explaining the algorithm validation procedure. (A) The algorithm was evaluated considering 29,250 evaluation runs ($n = 29,250$), corresponding to the number of different combinations between cells/particles/locations which contributed for generating the training and testing 2-seconds signal portions sets (13 "no particle" different locations $\times$ 15 different *Mock* cells $\times$ 15 different *HST6* cells $\times$ 10 different Polystyrene particles). According to this procedure, the classification algorithm was validated considering whether each particle is part of the training or test set. In each evaluation run, the particles selected for testing the classifier were never involved in the training. (B) After determining the most suitable combination between training parameters and training the classifier using these settings, test samples were classified considering this optimal training model (here we used the 29,250th evaluation run as an example).

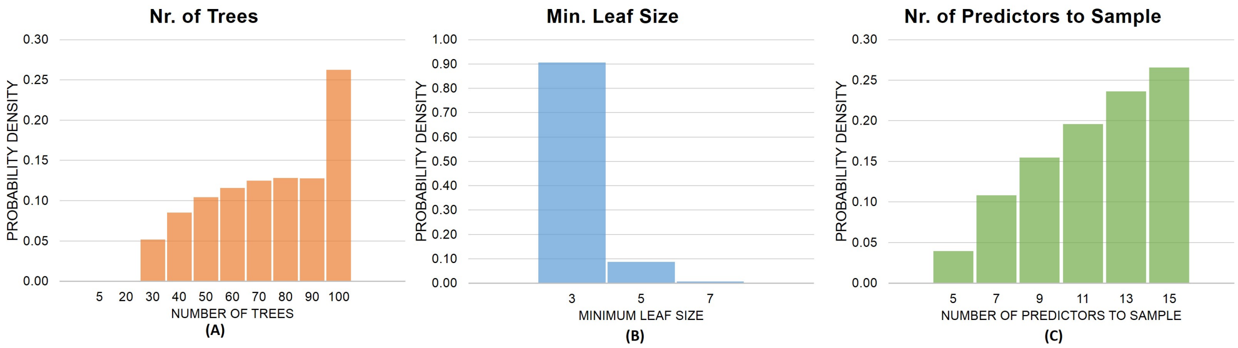

Figure S5: Probability density histograms representing how frequently each classifier parameter value was considered the most suitable for classes identification in the Cross-Validation procedure, along the different 29,250 different combinations between training and test sets: (A) number of trees considered; (B) minimum leaf size and (C) number of predictors to sample.

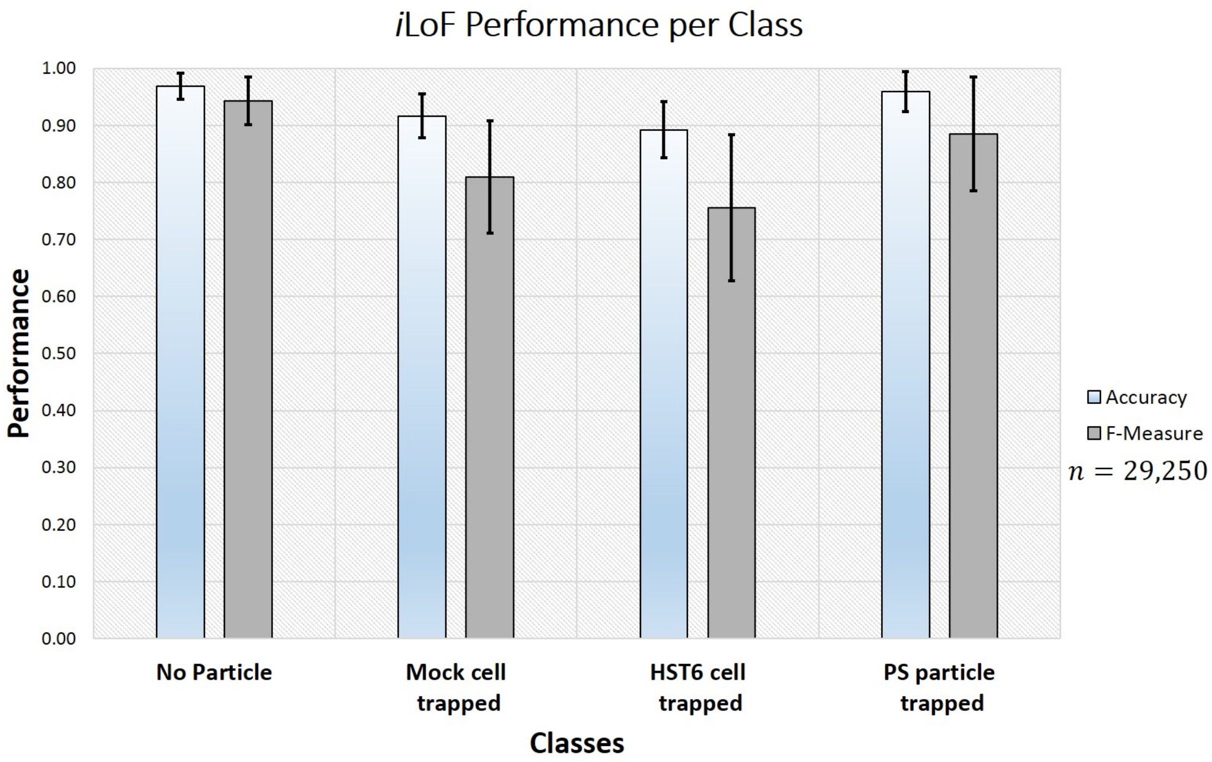

Figure S6: *i*LoF performance values per class considering the 29,250 evaluation runs. The error bars represent standard errors (for $N = 4$ classes).

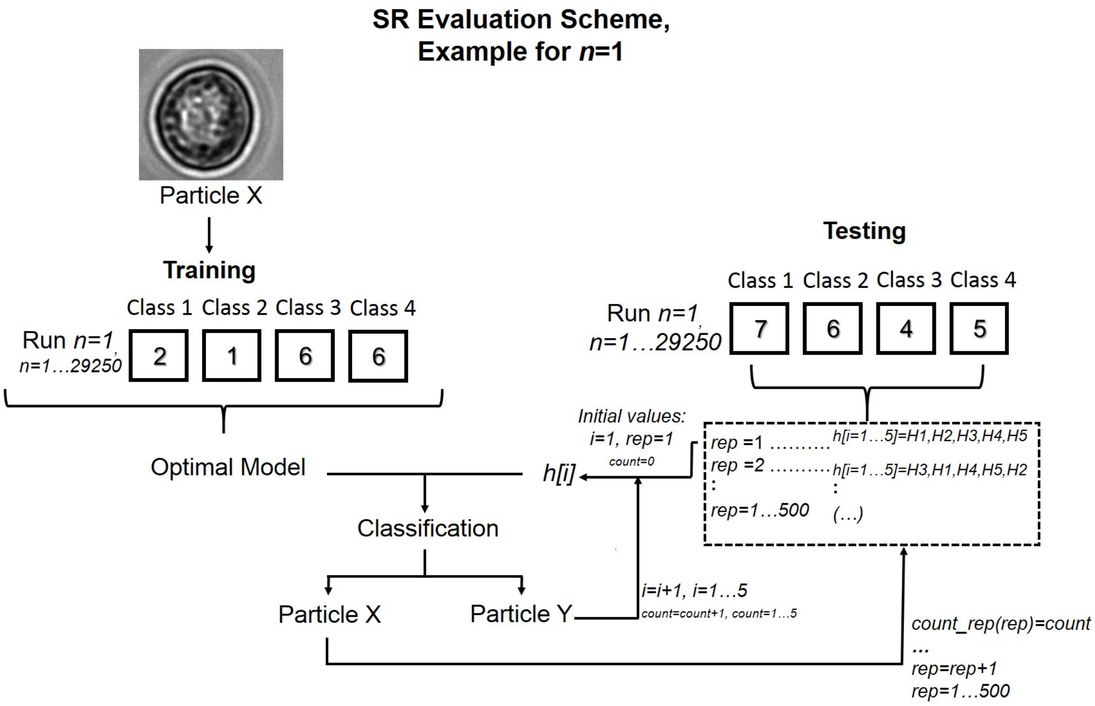
SR Evaluation Scheme,
Example for n=1
Particle X
Training
Class 1 Class 2 Class 3 Class 4
Run n=1,
n=1…29250
2 1 6 6
Optimal Model
Classification
Particle X
Particle Y
Testing
Class 1 Class 2 Class 3 Class 4
Run n=1,
n=1…29250
7 6 4 5
Initial values:
i=1, rep=1
count=0
h[i]
rep =1 ………h[i=1…5]=H1,H2,H3,H4,H5
rep =2 ………h[i=1…5]=H3,H1,H4,H5,H2
rep=1…500
(…)
i=i+1, i=1…5
count=count+1, count=1…5
count_rep(rep)=count
…
rep=rep+1
rep=1…500

Figure S7 *(preceding page)*: Scheme adopted to evaluate how many 2 seconds signal portions are necessary for a correct particles classification (corresponding to the SR of the proposed detection method) for evaluation run $n = 1$ as an example. Considering each particle, for each one of the *n* different combinations between the test and training sets: h represents the set of 2 s back-scattered signal portions that composes the test set. Supposing that h contains five signal portions in this example case, i represents the position of each signal portion of h in the test set; Hi represents the ith signal portion of the test set - for example $H1$ is the first signal portion of the test set - and *rep* is the pre-determined number of repetitions for which this procedure has to be run for each one of the *n* combinations. The variable *count* is incremented every time the algorithm fails to detect the particle using a given back-scattered signal portion, until the total number of signal portions is equal to those in the test set (in the example case illustrated in the figure, this number is 5). If the particle is wrongly identified using a given signal portion (for example $H1$ for the first repetition $rep = 1$), then the algorithm uses the information corresponding to the following signal portion - in this specific case, $H2$ - to identify the particle. If the particle is correctly identified or all the signal portions were already used separately in an attempt to identify the particle ($count = 50$, in this specific case), then the variable rep is incremented and the signal portions belonging to the test set are reordered - to ensure that the sequence of signal portions chosen to separately identify the particle is not the same as any one of those used in all 500 repetitions.

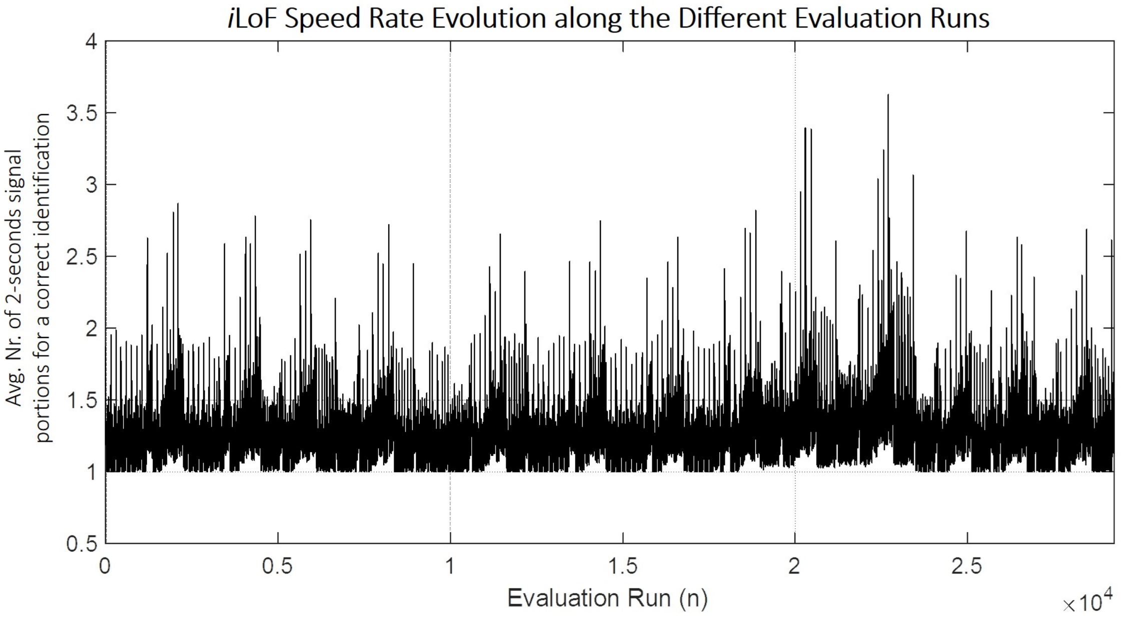

Figure S8: Mean number of 2-seconds signal portions needed for a correct class identification among the 500 times repetitions, for each evaluation run (n). Avg - average. Nr - number.

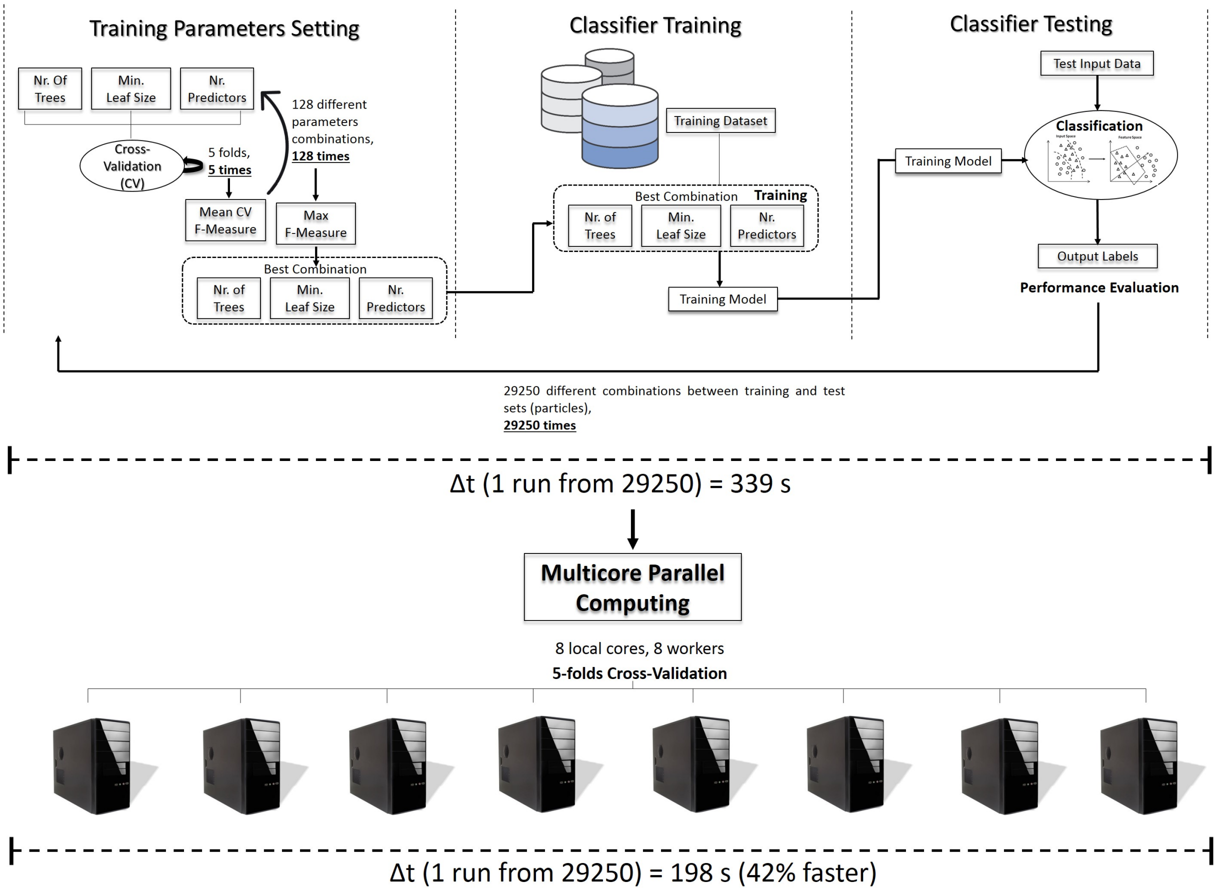
Training Parameters Setting
Nr. Of Trees
Min. Leaf Size
Nr. Predictors
128 different parameters combinations, 128 times
Cross-Validation (CV)
5 folds, 5 times
Mean CV F-Measure
Max F-Measure
Best Combination
Nr. of Trees
Min. Leaf Size
Nr. Predictors
Classifier Training
Training Dataset
Best Combination
Training
Nr. of Trees
Min. Leaf Size
Nr. Predictors
Training Model
Classifier Testing
Test Input Data
Training Model
Classification
Output Labels
Performance Evaluation
29250 different combinations between training and test sets (particles), 29250 times
Δt (1 run from 29250) = 339 s
Multicore Parallel Computing
8 local cores, 8 workers
5-folds Cross-Validation
Δt (1 run from 29250) = 198 s (42% faster)

Figure S9 *(preceding page)*: Scheme representing the complexity of the computational problem proposed here and how it was managed. Considering each one of the 29,250 different combinations between particles, the algorithm was run for five times within the five-fold Cross Validation method for each different combination between training parameters ($5 \times 180 = 900$ iterations), to determine the most suitable training parameters set (Training Parameters Setting stage). Then, the Random Forests classification algorithm was trained considering the optimal parameters previously determined and the training dataset to obtain a training model suitable for correct classification of novel input samples (Classifier Training phase). This model is therefore used to project the test features into the training features multidimensional space during the Classifier Testing phase. All these stages (Training Parameters Setting, Classifier Training and Classifier Testing) were run 29,250 iterations. To reduce computation time, considering each one of the 29,250 evaluation runs and each one of the 180 parameters set evaluated during Training Parameters Setting, we distributed the five iterations inherent to the 5-fold Cross-Validation for the eight cores of our machine using the Parallel Computing Toolbox from MATLAB$^{®}$, which led to a time reduction per evaluation run of 42 %.

Table S1: Identified *N*-linked glycans of *ST6* and *Mock* transfected cells. For each structure theoretical and detected mass of singly charged (sc) and doubly charged (dc) ion, retention time (rt) of elution, area under the peak (intensity) and relative abundance (%) are displayed.

Theoretical mass		Composition	Proposed structure	Detected mass		Mock			ST6GALNAC1			
sc	dc			sc	dc	rt (min)	Intensity	%	rt (min)	Intensity	%	**Ratio**
911,34	/	HexNAc2Hex3	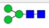	911,03	/	17,52-17,76	10391,2	0,14	17,51-17,76	7167,6	0,12	**0,89**
1057,4	/	HexNAc2Hex3Fuc1	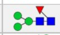	1057,09	/	23,30-23,64	67457,5	0,88	23,03-23,29	74470	1,25	**1,42**
1235,44	/	HexNAc2Hex5	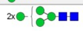	1235,07	/	21,57-21,91	146487,5	1,91	21,34-21,68	100866	1,69	**0,88**
1397,5	698,24	HexNAc2Hex6	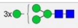	1397,08	698,31	19,29-19,60	1226141	16,01	19,30-19,52	904836,4	15,18	**0,95**
1559,55	779,27	HexNAc2Hex7	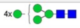	1559,03	779,17	18,78-19,07	1314644	17,17	18,78-19,07	1047276	17,57	**1,02**
1713,62	856,31	HexNAc3Hex4Sia1Fuc1	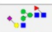	1713,12	856,83	23,72-24,13	341831,4	4,46	23,29-23,70	232156,2	3,89	**0,87**
1721,6	860,3	HexNAc2Hex8	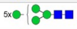	1721,02	860,3	18,93-19,21	1644482	21,47	18,86-19,15	1457053	24,44	**1,14**
/	864,3	HexNAc3Hex5Sia1	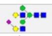	/	864,12	21,16-21,49	97387	1,27	20,93-21,26	55601,5	0,93	**0,73**
/	937,34	HexNAc3Hex5Sia1Fuc1	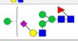	/	937,19	23,05-23,39	83674	1,09	22,69-23,03	68687	1,15	**1,05**
/	941,33	HexNAc2Hex9	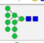	/	941,09	19,14-19,44	645903	8,43	19,07-19,38	526569,5	8,83	**1,05**
/	945,33	HexNAc3Hex6Sia1	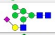	/	945,05	22,46-22,71	164440	2,15	22,01-22,35	114456	1,92	**0,89**
/	965,84	HexNAc4Hex5Sia1	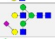	/	965,61	22,54-23,05	153594	2,01	22,10-22,69	105002,4	1,76	**0,88**
/	1022,35	HexNAc2Hex10	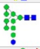	/	1022,1	19,93-20,18	112636,8	1,47	19,77-20,02	87682	1,47	**1,00**
/	1038,87	HexNAc4Hex5Sia1Fuc1	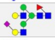	/	1038,52	24,38-24,79	315053,4	4,11	23,96-24,37	273725,4	4,59	**1,12**
/	1111,39	HexNAc4Hex5Sia2	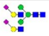	/	1111,07	24,88-25,29	281691,6	3,68	24,46-25,14	125333,1	2,10	**0,57**
/	1111,39	HexNAc4Hex5Sia2	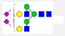	/	1111,12	28,14-28,73	183112,8	2,39	27,85-28,87	121747,6	2,04	**0,85**
/	1184,42	HexNAc4Hex5Sia2Fuc1	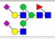	/	1184,08	26,47-26,88	630633,6	8,24	26,07-26,75	380829,6	6,39	**0,78**
/	1184,42	HexNAc4Hex5Sia2Fuc1	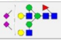	/	1184,06	29,56-30,15	238402,4	3,11	29,38-30,31	278018,4	4,66	**1,50**
**Total:**							7657963	100		5961478	100	

Table S2: Identified *O*-linked glycans of *ST6* and *Mock* transfected cells. For each structure theoretical and detected mass of singly charged (sc) and doubly charged (dc) ion, retention time (rt) of elution, area under the peak (intensity) and relative abundance (%) are displayed.

Theoretical mass		Composition	Proposed structure	Detected mass		Mock			ST6GALNAC1			
sc	dc			sc	dc	rt (min)	Intensity	%	rt (min)	Intensity	%	**Ratio**
384,15	/	HexNAc1Hex1	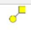	384,09	/	5,64-6,41	33872,4	1,3%	5,02-5,55	57664	2,3%	**1,74**
513,19	/	HexNAc1Sia1	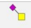	513,09	/	11,95-12,18	5238,8	0,2%	14,27-14,82	61897,6	2,5%	**12,09**
587,23	/	HexNAc2Hex1	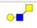	587,11	/			0,0%	18,64-19,11	19113,5	0,8%	
675,25	/	HexNAc1Hex1Sia1	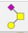	675,11	/	13,59-14,07	80399,9	3,2%	16,06-16,61	168817,6	6,8%	**2,15**
675,25	/	HexNAc1Hex1Sia1	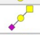	675,12	/	16,25-16,79	288041,6	11,3%	18,72-19,27	285753,6	11,5%	**1,01**
733,29	/	HexNAc2Hex1Fuc1	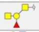	733,07	/	16,95-17,11	7194	0,3%	20,12-20,27	10173,6	0,4%	**1,45**
749,28	/	HexNAc2Hex2	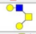	749,09	/	15,63-15,79	6359,4	0,2%	18,80-19,11	63956	2,6%	**10,29**
749,28	/	HexNAc2Hex2	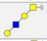	749,09	/	16,48-16,64	5402,7	0,2%	19,65-19,81	15597,3	0,6%	**2,95**
749,28	/	HexNAc2Hex2	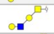	749,09	/	16,95-17,11	15933,3	0,6%	20,12-20,35	23124,4	0,9%	**1,48**
878,36	/	HexNAc2Hex1Sia1	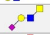	878,11	/	24,81-25,38	27052,8	1,1%	26,47-26,80	49573	2,0%	**1,87**
895,34	/	HexNAc2Hex2Fuc1	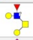	895,08	/	13,28-13,59	3933	0,2%	16,53-16,85	14475	0,6%	**3,77**
966,34	/	HexNAc1Hex1Sia2	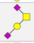	966	/	20,08-21,28	375865,6	14,8%	21,28-22,23	191331,4	7,7%	**0,52**
1040,38	/	HexNAc2Hex2Sia1	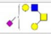	1040,08	/	21,51-22,00	60053,7	2,4%	23,51-23,99	168144,9	6,8%	**2,86**
1040,38	/	HexNAc2Hex2Sia1	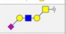	1040,05	/	22,48-23,22	132493	5,2%	24,32-24,72	56116,8	2,3%	**0,43**
1040,38	/	HexNAc2Hex2Sia1	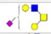	1040	/	24,56-24,65	34399,6	1,4%	25,99-26,39	156181,8	6,3%	**4,64**
1114,42	/	HexNAc3Hex3	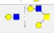	1114,09	/	20,24-20,55	5890,5	0,2%	22,96-23,28	30929	1,2%	**5,37**
1331,47	665,23	HexNAc2Hex2Sia2	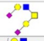	/	665,13	28,57-29,28	154088	6,1%	29,77-30,50	160701	6,5%	**1,07**
2061,74	1030,37	HexNAc4Hex4Sia2	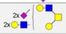	/	1030,13	33,41-36,42	700233,6	27,5%	33,56-35,22	471209,2	18,9%	**0,69**
2426,87	1212,93	HexNAc5Hex5Sia2	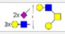	/	1212,45	34,79-37,72	610155,9	24,0%	34,44-38,07	484536	19,5%	**0,81**
**Total:**							2546608	100%		2489296	100%	

Table S3: Optical and morphological characteristics of the particles samples used in this study. n - Refractive Index. Nr. - number.

**Solutions ID**	**Solvent**	**Particles/Cells Type**	**Particles/Cells Diameter**	**Nr. of Particles/Cells**
Solution 1		*Mock* cells	15.6 ± 2.9	15
Solution 2	PBS (n=1.360)	*HST6* cells	16.2 ± 3.1	15
Solution 3		Polystyrene particles	8.0 ± 1.6	10

Table S4: Dataset characterization after signal processing. Each sample corresponds to each 2-seconds back-scattered signal portion. Nr - number. Avg - average. *The "No particle" condition was treated in the same way as a type of microparticle. For this class in particular, each particle corresponds to different acquisitions performed with no particle in front of the tip.

**Class**	**1: "No Particle"**	**2: "*Mock* cell trapped"**	**3: "*HST6* cell trapped"**	**4: "PS particle trapped"**
Nr. of Particles*	13	15	15	10
Nr. of signal portions per particle	34 ± 3	32 ± 5	31 ± 5	31 ± 6
Total nr. of signal portions	438	479	470	305

Table S5: List of parameters tuned during classifier training stage for model optimization. Nr. - Number. Min. - Minimum.

**Training Parameters**	
**Nr. of Trees**	5, 20, 30, 40, 50, 60, 70, 80, 90, 100
**Min. Leaf Size**	3, 5, 7
**Nr. Predictors To Sample**	5, 7, 9, 11, 13, 15
**Nr. of Different Parameters Combination**	10 x 3 x 6 = 180

Table S6: Training and test dataset characterization along the 29,250 different evaluation runs. Each dataset sample corresponds to a 2-seconds back-scattered signal portion. Avg - average. Nr - number.

		$n = 29,250$	
		**Avg. Nr. of Samples Per Particle**	**Avg. Total Nr. of Samples**
**Train**	Class 1: "No Particle"	403 ± 3	1564 ± 9
	Class 2: "*Mock* Cell"	447 ± 5	
	Class 3: "*HST6* Cell"	439 ± 5	
	Class 4: "PS Particle"	275 ± 5	
**Test**	Class 1: "No Particle"	34 ± 3	127 ± 9
	Class 2: "*Mock* Cell"	32 ± 5	
	Class 3: "*HST6* Cell"	31 ± 5	
	Class 4: "PS Particle"	31 ± 5	

Supplementary Note S1: *i*LoF Speed Detection Rate

Overall, the *i*LoF device is characterized by an average SR of 1.17 ± 0.51 2-seconds short-term signal portions, totaling 2.3 ± 1.0 seconds (fig. **??**.(B)). However, more details about the *i*LoF SR of detection can be extracted from figs. **??** and S8.

In 10% of the SR evaluation runs, two signal portions (4 seconds) were necessary for a correct identification by the *i*LoF. However, the percentage of times in which the *i*LoF was able to correctly classify the type of particle trapped by using more than 2 signal portions was almost residual (fig. **??**.(A)), over the 14,750,000 SR test runs. Considering the evolution of the mean 2-seconds signal portions needed for a 100 % accuracy for each one of the 24,250 different combinations (Supplementary fig. S8), the *i*LoF SR varies between one and 3.62 ± 2.79 $(n = 500)$ signal portions (corresponding to 2 seconds and 7.24 ± 5.58 seconds, respectively). Even considering the combination between training and test sets for which the worst performance in terms of SR was obtained, the *i*LoF needed in average of 3.62 ± 2.79 portions $(n = 500)$ (Supplementary fig. S8).

Videos: Video S1

Video showing the stable 2D optical trapping of a *HST6* cancer cell using the fabricated microlens-like structure on the top of an optical fiber.

1. Ribeiro, R., Soppera, O., Oliva, A., Guerreiro, A. & Jorge, P. New trends on optical fiber tweezers. *Journal of Lightwave Technology* **33**, 3394–3405 (2015).
